# Vesicle adhesion in flow

**DOI:** 10.1140/epje/s10189-026-00628-1

**Published:** 2026-09-22

**Authors:** Lucia Wesenberg, Felix Weissenfeld, Kai-Uwe Hollborn, Motomu Tanaka, Marcus Müller

**Affiliations:** 1 Institut für Theoretische Physik, Georg-August-Universität Göttingen, Friedrich-Hund-Platz 1, 37077 Göttingen, Germany; 2 Physical Chemistry of Biosystems, Institute of Physical Chemistry, Heidelberg University, 69120 Heidelberg, Germany; 3 Center for Integrative Medicine and Physics, Graduate School of Medicine, Kyoto University, Kyoto, 606-8502 Japan

## Abstract

**Abstract:**

The dynamic adhesion of lipid vesicles in shear flow is governed by the interplay of membrane-bending rigidity, membrane–substrate adhesion energy, and hydrodynamic stresses. While the equilibrium contact curvature is determined by the transversality condition, shear flow can additionally deform the adhered vesicle, drive translational motion, or, through hydrodynamic lift forces, promote detachment of the vesicle from the substrate. Here, we investigate adhered vesicles in shear flow subjected to a reversible change of the membrane-wall interactions. Using a stimuli-responsive poly(acrylic acid-co-cysteine) brush, whose adhesive properties are modulated by the controlled addition and removal of Cd$^{2+}$ ions, and using reflection interference contrast microscopy, we track the membrane–substrate separation and vesicle shape throughout the transition. Using molecular dynamics simulations within the Helfrich framework, we provide microscopic insights into the deformation processes and systematically investigate the dependence on the shear-flow strength. Two parameters govern the dynamic response: (i) the balance between adhesion and membrane bending, and (ii) the ratio between viscous shear and bending stresses. In the strong-adhesion regime, a large rim of the contact zone between membrane and substrate and an increased density of wall springs lead to a significant increase in wall springs that must rupture per unit displacement. This effectively suppresses tank-treading motion. Reducing adhesion decreases the contact-zone rim and wall-spring density, enabling flow-driven translational transport. Simultaneously, shear flow induces a shape asymmetry, manifested as a forward tilt of the vesicle. As a consequence, the curvature at the preceding edge decreases, whereas the curvature at the receding edge increases. These results demonstrate that vesicle motion on polymer-brush-coated substrates can be reversibly controlled by chemical stimuli, providing a tunable platform to study adhesion dynamics under flow.

**Graphical abstract:**

**Figure:**
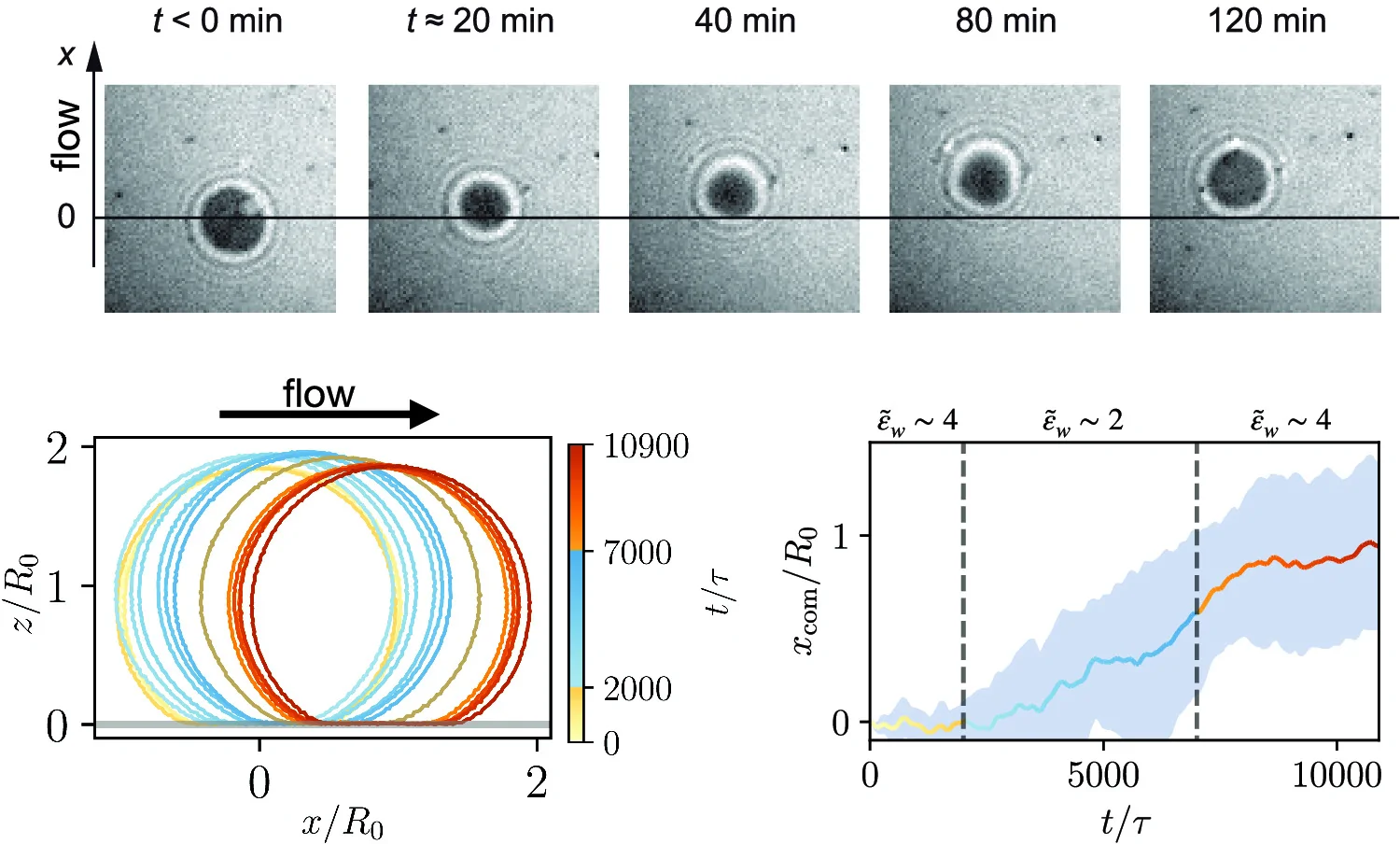

**Supplementary Information:**

The online version contains supplementary material available at https://doi.org/10.1140/epje/s10189-026-00628-1.

## Introduction

The wetting of solid substrates by thin liquid films is both ubiquitous and fundamentally important across the life sciences, natural sciences, and engineering. Simultaneously, it continues to pose significant theoretical challenges [1–5]. A macroscopic droplet of fixed volume adopts the shape of a spherical cap characterized by a contact angle, Θ, determined by Young’s equation [6]. This relation balances the difference in liquid-substrate and vapor-substrate tensions, $\Delta \gamma _\textrm{w}$, against the liquid–vapor interfacial tension, $\gamma _\textrm{LV}$. Importantly, this balance is independent of droplet size, enabling the extraction of macroscopic wetting properties from molecular simulations of microscopic systems [7, 8].

When a droplet is driven by an external body force, its dynamics are governed by several, distinct scale-dependent dissipation mechanisms [9–13]: (i) viscous dissipation within the liquid bulk, (ii) friction arising from liquid slip past the solid substrate, and (iii) dissipation localized at the three-phase contact line between solid, liquid, and vapor.

Lipid membranes—a fundamental structural component of biological cells—are approximately 5 nm-thick viscoelastic bilayer films capable of self-assembly into closed vesicles. In contrast to wetting of liquid droplets, the adsorption of a vesicle with fixed membrane area onto a solid substrate is governed by the competition between the surface tension difference, $\Delta \gamma _\textrm{w}$, and the membrane-bending rigidity, κ. This local free-energy balance depends explicitly on the vesicle size, $R_0$, through the dimensionless parameter

1 $$\begin{aligned} \tilde{\varepsilon }_w = \frac{\Delta \gamma _\textrm{w}R_0^2}{\kappa } \end{aligned}$$

As a result, vesicle adhesion can be tuned through the vesicle size itself, rather than by changing the chemistry of the membrane or substrate—in marked contrast to droplet wetting, where no equivalent size dependence exists. This balance can be formulated as a transversality condition [14, 15], which determines the maximal contact curvature at the rim of the adhesion zone,

2 $$\begin{aligned} C^2_{\max } = \frac{2\Delta \gamma _\textrm{w}}{\kappa } \end{aligned}$$

In marked contrast to the intrinsic, thermodynamic liquid–vapor surface tension of a droplet, the membrane tension is not a material constant but a Lagrange multiplier enforcing the area constraint; it therefore depends on the global membrane geometry [15–17].

Additional physical effects modify the adhesion-bending balance, Eq. 2. Buoyancy and a finite interaction range between the substrate and the membrane lead to quantitative corrections [18, 19]. More fundamentally, any finite-range membrane–substrate interaction potential, *V*(*z*) (with *z* denoting the distance normal to the substrate), induces a first-order adsorption transition at $\Delta \gamma _\textrm{w} = 0$ [18, 19]. A continuous (second-order) adsorption transition emerges only in the singular limit of zero-range interactions, at $\tilde{\varepsilon }_\textrm{w}^* = 2$ [14, 15, 18, 19].

The membrane–substrate interaction, $\Delta \gamma _\textrm{w}$, can be reversibly tuned by coating the solid support with a stimuli-responsive polymer brush—a strategy that has also been successfully applied to switch the wettability and shape of liquid droplets [13, 20, 21]. Specular neutron and X-ray reflectivity studies of polymer-supported membranes have shown that the thickness of the interfacial water layer between the membrane and the brush is governed by a balance of attractive van-der-Waals forces, depletion attraction, short-range hydration repulsion, and Helfrich undulation repulsion [22]. Stimuli-responsive polymer brushes alter their physicochemical properties, such as chain conformation, degree of ionization, and solvent affinity, in response to external triggers including temperature, light, pH, or ionic strength [23–25]. In the present work, we reversibly switch the membrane–substrate interaction through the addition and removal of Cd$^{2+}$ ions in a stimuli-responsive polymer brush composed of linear poly(acrylic acid) with cysteine side chains (pAA-Cys5) [26, 27].

Understanding the dynamics of vesicle adsorption has attracted abiding interest. Lipid membranes can spread over macroscopic substrate areas of several tens of cm$^2$ [28, 29]. Notably, these biomolecular films possess self-healing capabilities, enabling them to repair local defects [30]. The mechanisms by which vesicles spread on solid substrates—specifically, whether the inner or outer monolayer ultimately contacts the surface—are sensitive to the range and form of the membrane–substrate interaction potential, *V*(*z*) [31].

In this study, we investigate distinct wetting states of a vesicle adhering to a stimuli-responsive polymer brush under shear flow. Shear flow introduces an additional dimensionless characteristic—the capillary number [32–34]

3 $$\begin{aligned} \textrm{Ca} = \frac{\dot{\gamma } \eta R_0^3}{\kappa } = \dot{\gamma } \tau _\textrm{shape}\,, \end{aligned}$$

which quantifies the ratio of viscous stress to bending stress and thereby governs flow-induced vesicle deformation. $\tau _\textrm{shape} \equiv \eta R_0^3/\kappa$ quantifies the shape-relaxation time of the vesicle. Furthermore, shear flow can drive translational motion and, through hydrodynamic lift forces, promote detachment of the vesicle from the substrate.

The dynamic change in wetting states was monitored as the modulation of the fluctuation of the membrane–substrate distance and the change in vesicle shape near the substrate by using reflection interference contrast microscopy (RICM). Beyond controlling adhesion, polymer brushes also modulate the frictional coupling between the membrane and the solid support [35, 36].

In parallel, we model the lipid membrane as a thin elastic sheet within the Helfrich framework [16], enabling us to reproduce the experimental observation in simulations and to extend our analysis to nonequilibrium steady states of adhered vesicles in shear flow.

## Materials, methods and models

### Materials

For all experiments, Milli-Q water from an ultra-purification system (Merck Millipore, Darmstadt, Germany) with a resistance > 18 MΩ cm was used. All lipids, 1,2-dioleoyl-*sn*-*glycero*-3-phosphocholine (DOPC), 1,2-dioleoyl-*sn*-*glycero*-3-phosphoethanol amine- *N*-(cap biotinyl) (DOPE-biotin), and Texas Red™ 1,2-dihexadecanoyl-*sn*-glycerin-3-phosphoethanolamine (DHPE-Texas Red), were obtained from Avanti Polar Lipids (Alabama, USA). Neutravidin from Thermo Fisher Scientific (Karlsruhe, Germany) was ultracentrifuged before use (100,000 g, 1 h). For the experiments, the supernatant was used. All other chemicals were purchased from Sigma-Aldrich (Taufkirchen, Germany) unless stated otherwise.

The pAA-Cys5 polymer was synthesized by reversible addition-fragmentation chain transfer (RAFT) polymerization as described in the previous accounts [18, 26, 27]. The molecular weight was estimated to be $M_{\text {W}}$ = 74 kDa.

Microfluidic experiments were conducted in a custom-made (bottomless) μ-Slide III 3 in 1 channel from ibidi (Gräfelfing, Germany), having a channel width of 3 mm and a height of 0.4 mm. The bottom was replaced by a microscope slide.

### Methods

#### Preparation of lipid vesicles

Lipids dissolved in chloroform (1 mg mL$^{-1}$) containing 98 mol% DOPC and 2 mol% DOPE-biotin were kept in a vacuum oven overnight to remove chloroform residues. The dried lipids were resuspended in a 100 mM NaCl buffer at pH = 7.4, buffered with 10 mM Tris. To obtain small unilamellar vesicles (SUVs), the lipid suspensions were sonicated with a Misonix Sonicator 3000 (Misonix, Düsseldorf, Germany). Giant unilamellar vesicles (GUVs) were prepared by following the electroswelling method according to the previous account[18]. Briefly, a lipid mixture of DOPC containing 0.2 mol% DHPE-Texas Red was deposited onto indium tin oxide (ITO) coated glass slides (Sigma-Aldrich) by spin coating. The dried lipids were hydrated in a 300 mM (≈300 mOsm/kg H_2_O) sucrose solution. In a custom-built chamber, an AC current of 3 V with a frequency of 10 Hz was applied for 2 h at 37 °C. To enable adhesion of vesicles, the GUVs were suspended in a slightly less dense outer solution, e.g., a 340 mM glucose solution containing 10 mM Tris buffer containing 1 mM cadmium (≈360 mOsm/kg H_2_O) or a 310 mM glucose solution containing 10 mM EDTA buffered with 10 mM Tris buffer (≈360 mOsm/kg H_2_O). The osmolality was monitored by using an OM 806 micro-osmometer (Löser, Berlin, Germany). This osmolality difference (up to 60 mOsm/kg H_2_O) remains comparable to the conditions taken in the previous experimental accounts [18, 37–39].

#### Fabrication of switchable polymer-coated substrates

Switchable substrates were fabricated according to the previous account [18]. Briefly, a supported lipid bilayer was deposited by incubating a glass slide with small unilamellar vesicles (SUVs) containing 98 mol% DOPC and 2 mol% DOPE-biotin for 30 min at 40°C. Non-fused SUVs were removed by rinsing with buffer. Next, the sample was incubated with neutravidin (40 μg mL$^{-1}$) for 1 h at 40°C. Unbound neutravidin was removed, and the sample was subsequently incubated with pAA-Cys5 (40 μg mL$^{-1}$) under identical conditions. In the next step, the buffer was switched to glucose 340 mM buffered with 10 mM Tris containing 1 mM CdCl$_2$. Finally, a portion of giant unilamellar vesicles was deposited. After allowing the GUVs to sediment, the flow was induced at the respective flow rates.

#### Microinterferometry

To track vesicle movement and extract the adhesion area and vesicle shape in the vicinity of the substrate surface, the vesicles were monitored using reflection interference contrast microscopy (RICM). RICM imaging was carried out on an Axio Observer Z1 microscope (Zeiss, Oberkochen, Germany) equipped with a 63 × oil immersion objective (NA 1.25) and a built-in quarter-wave plate. Every Δ t = 1 min, RICM images were collected at an exposure time of 30 ms. Using ImageJ, the adhesion area was fitted with an ellipse, and the center of mass was determined.

#### Shape analysis

RICM enables the reconstruction of the height profile *h* of GUVs near the substrate [18]. The periodic ambiguity of the intensity-height relation [40] is resolved by separating the ascending and descending branches of the intensity profile while taking the fringe sequence into account [41, 42]. The local height of membrane *h*($I_x$) can be calculated,

4 $$\begin{aligned} h(I_x) = m\Delta h + I_{\text {norm}}\frac{\Delta h}{2}, \end{aligned}$$

where *m* is the fringe order and Δh = $\lambda _{\text {RICM}}$/2n is the height difference between the intensity maximum and minimum. $I_{\text {norm}}$ is the normalized intensity,

5 $$\begin{aligned} I_{\text {norm}} = \frac{I_x-I_{\text {min}}}{I_{\text {max}}-I_{\text {min}}}. \end{aligned}$$

Note, *m* = 0 corresponds to the first minimum. The calculations were performed by using a self-written routine in Igor Pro (Wavemetrics, Lake Oswego, USA).

### Simulation model

**Fig. 1:**
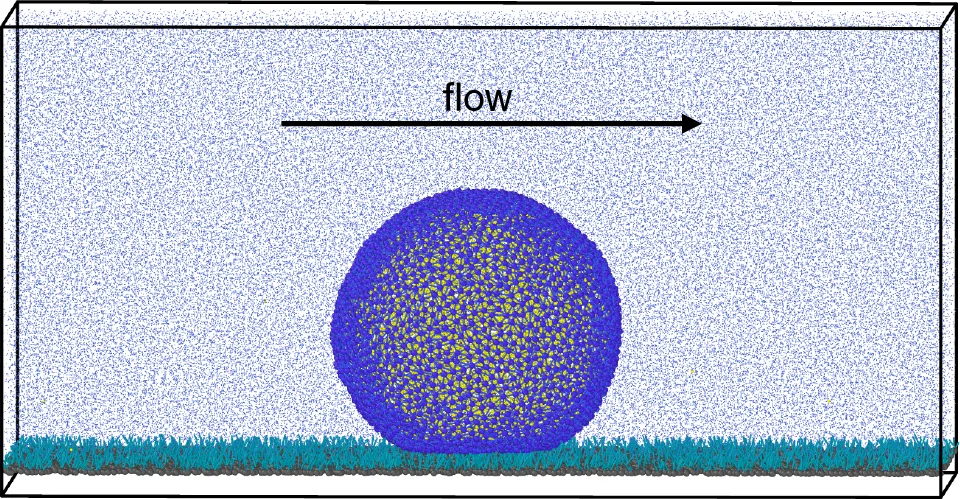
Schematic illustration of the adhered vesicle model under flow. The vesicle membrane is represented by a triangulated mesh composed of vertices and connecting edges (blue). Adhesion to the substrate is mediated by an imposed adhesion potential that diverges at the positions of fixed wall particles (gray). These wall particles also serve as anchoring points for wall springs (turquoise), which effectively impose a no-slip boundary condition between the solvent/membrane and the substrate. The vesicle is filled with one solvent (yellow) and surrounded by a second solvent (blue), and the solvent and vesicle interact via a Dissipative-Particle-Dynamics (DPD) potential. Collectively, the adhesion potential, DPD interactions, and wall springs govern the equilibrium adhesion and nonequilibrium dynamical response of the vesicle–substrate system

In our simulations, we investigate the dynamics of a deformable vesicle in shear flow, adhered to a planar substrate with tunable adhesion strength and local adsorption/desorption dynamics at the rim of the contact zone (Fig. 1). The vesicle membrane is represented as a fluid, triangulated surface [16, 43, 44] in an explicit solvent. Hydrodynamics is implemented by a momentum-conserving Dissipative Particle Dynamics (DPD) thermostat [45, 46]. The membrane–substrate interactions include both an attractive-repulsive potential and wall springs [47] to account for adhesion, and tailor the local adsorption/desorption dynamics. All model components are shortly introduced below; additional details are provided in Sec. S1 of the supplementary material.

#### Vesicle membrane model

The vesicle membrane is modeled as a fluid, triangulated surface [43, 44] consisting of $N_\textrm{vert}$ vertices connected by a dynamically reconfiguring network of bonds. Its bending energy is given by the discretized Helfrich Hamiltonian [16],

6 $$\begin{aligned} \mathcal {H}_{\textrm{bend}} = \frac{\kappa }{2}\sum _{i} \left( C_i - C_0 \right) ^2 A_i, \end{aligned}$$

where κ is the bending rigidity, $C_i$ denotes the local mean curvature at vertex *i*, $C_0$ is the spontaneous curvature (set to zero in this work), and $A_i$ is the associated vertex area. Details of the discretized curvature operator can be found in Refs. [48, 49]. To ensure numerical stability and prevent unphysical bond stretching or collapse, neighboring vertices interact via an attractive–repulsive bond potential [44, 50, 51], which also effectively constrains the total membrane area.

Membrane fluidity is maintained by Monte Carlo bond-flip moves [44, 50]. At fixed intervals, the molecular dynamics integration is paused, and randomly selected bonds are proposed to flip, connecting the previously unconnected vertices of the two adjacent triangles.

The membrane is embedded in an explicit solvent, interacting via DPD interactions with a high repulsion parameter of $A_\mathrm{sol-ves} =60.0 \ k_BT/r_c^\textrm{DPD}$. By assigning membrane–solvent interactions a higher repulsion strength than solvent–solvent interactions, we effectively render the membrane impermeable on short timescales. All simulations were performed at a reduced volume of $v=V/V_0 \sim 1$.^1^

To model an experimentally motivated density mismatch between the vesicle interior and exterior, we can additionally apply a downward buoyancy force to the vesicle. To this end, a constant force is applied to all solvent particles enclosed within the vesicle,

7 $$\begin{aligned} \textbf{F}^{\textrm{buoy}}_{i} = -\Delta m g \hat{\textbf{z}} \end{aligned}$$

where $\hat{\textbf{z}}$ is the substrate normal, $\Delta m = m_\textrm{in} - m_\textrm{out}$ denotes the mass difference between the solvent particles of the interior and exterior fluids, *g* is the gravitational acceleration. Here, we have assumed that the number densities of the interior and exterior fluids are identical. Unless stated otherwise, no buoyancy is applied $\tilde{\varepsilon }_B = \frac{\Delta \rho g R_0^4 }{\kappa }= 0$. A nonzero buoyancy of $\tilde{\varepsilon }_B=-35$ is used only in subsection 3.7, where it counteracts shear-induced lift and induces a sedimentation of the vesicle toward the substrate.

#### Substrate interactions

The vesicle interacts with a planar substrate located at $z = z_\textrm{wall}$. Adhesion is controlled via a Mie-type wall potential acting on each membrane vertex,

8 $$\begin{aligned} U_{\textrm{wall}}(z) = \Delta \gamma _\textrm{wall} \left[ \left( \frac{\sigma _w}{z - z_\textrm{wall}} \right) ^9 - \left( \frac{\sigma _w}{z - z_\textrm{wall}} \right) ^3 \right] , \end{aligned}$$

where $\Delta \gamma _\textrm{wall}$ sets the bare adhesion strength and $\sigma _w$ defines the interaction range.

#### Local adsorption/desorption dynamics and flow

Wall springs dynamically form and break between membrane vertices and immobile anchoring points on the substrate. Together with $U_{\textrm{wall}}$, they model the adhesive contacts between the vesicle membrane and the pAA-Cys5 brush, whose strength is modulated by Cd$^{2+}$ ions. Springs between solvent particles and wall anchoring points impose a no-slip boundary condition ($v_x|_\textrm{wall} \approx 0$) [47]. Each wall spring is described by a finitely extensible nonlinear elastic (FENE) potential,

9 $$\begin{aligned} U_{\textrm{FENE}}(r) = -\frac{1}{2} k_\textrm{sp} r_{\max }^2 \ln \left( 1 - \frac{r^2}{r_{\max }^2}\right) , \end{aligned}$$

where $k_\textrm{sp}$ is the spring constant, *r* is the distance between the membrane vertex/solvent and anchoring point, and $r_{\max }$ is the maximal extension. Spring formation is attempted when a membrane vertex or solvent particle is within the cutoff distance of the anchoring points. The formation and rupture of wall springs are treated within the grand-canonical ensemble at a fixed activity $z^{ves}_{wlsp}$, which controls the mean number of wall springs $\langle N_\textrm{wlsp} \rangle$. Both moves are accepted according to the Metropolis criterion based on the change in FENE energy; thus, stretched springs are more likely to rupture. Due to the FENE potential, the wall springs introduce an additional attraction between the membrane/solvent and the substrate, which is exactly canceled by a compensating potential acting between all particles that can potentially be connected by wall springs [47], thereby decoupling adhesion free energy and local adsorption/desorption dynamics.

Poiseuille flow is generated by applying a constant body force in the *x*-direction to solvent particles and membrane vertices,

10 $$\begin{aligned} \textbf{F}^{\textrm{flow}}_i = F_0 \hat{\textbf{x}}, \end{aligned}$$

where $F_0$ sets the flow strength. This body-force-driven protocol establishes a Poiseuille-like velocity profile in the viscous fluid, which gives rise to an approximately constant shear rate, $\dot{\gamma }$, in the vicinity of the substrate. In the Stokes regime, a uniform body force is equivalent to an imposed pressure gradient, $\nabla p = -\rho F_0 \hat{\textbf{x}}$, which acts on all fluid particles.

The dynamic viscosity η of the DPD solvent was determined from the flow profile of pure solvent simulations, yielding $\eta \approx 0.97 \ k_{B} T \tau /(r_c^\textrm{DPD})^3$, where $r_c^\textrm{DPD}$ is the DPD cutoff radius (for details see subsection S2.1).

#### Sliding or tank-treading

**Fig. 2:**
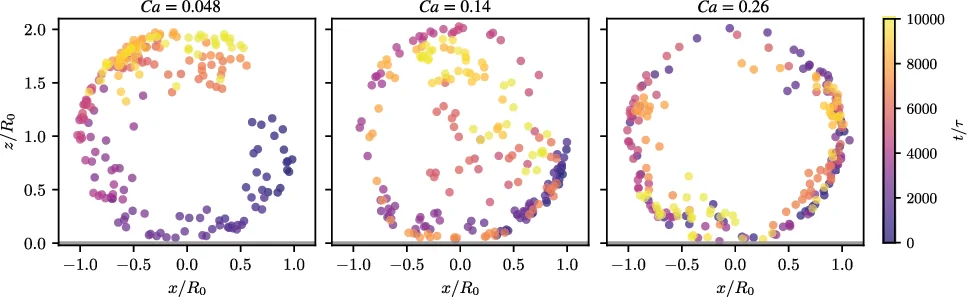
Trajectory of the vertex initially closest to y=0 in the center-of-mass frame of reference, presented for three driving forces $F_0 \in \{0.00002, 0.00006, 0.00011 \} k_B T/ r_c^\textrm{DPD}$ and resulting Capillary numbers Ca $\in \{ 0.048, 0.14, 0.26 \}$. Color indicates simulation time. The vertex circulates around the vesicle periphery, indicating tank-treading motion. Pure sliding would produce a stationary vertex position

For both weakly and strongly adhered vesicles in shear flow, tank-treading has been reported as the dominant mode of motion [52–54]. We investigated whether our vesicles exhibit tank-treading or sliding behavior by tracking individual membrane vertices.

Figure 2 presents the trajectory of the vertex, after removing the center-of-mass motion for three flow speeds. The color code indicates the simulation time. For purely sliding motion, one would expect the vertex position to remain fixed. Instead, in the center-of-mass reference frame, the membrane vertex continuously moves along the vesicle contour. In the laboratory frame, the vesicle therefore rolls over the substrate via a tank-treading motion.

To further substantiate this observation, we measured the *x*-direction velocity of the vertices closest to the substrate for trajectories with different flow and adhesion strengths. Under all conditions studied, $v_x|_\textrm{wall} \approx 0 r_c^{DPD} /\tau$ (cf. Figure S3a). Thus, tank-treading is the only observed mode of motion.

Note that during tank-treading, the rim of the contact zone between the vesicle and the substrate continuously attaches at the preceding edge and detaches at the receding edge. These dynamics are governed by the wall springs.

#### Simulation protocol

The temperature is controlled by a DPD thermostat [45, 46]. All simulations were performed with HOOMD-blue [55] together with custom plug-ins, implementing the membrane and wall–spring interactions. The equations of motion were integrated using the Velocity-Verlet algorithm with a timestep $\Delta t = 0.001\tau$, ensuring stable membrane dynamics and reliable temperature control, with $\tau = \sqrt{m(r^{DPD}_c)^2/k_B T}$. Bond-flip and wall-spring Monte Carlo updates were attempted every $N_\textrm{conn}$ and $N_\textrm{wlsp}$ MD steps, respectively. A complete list of simulation parameters is provided in Sec. S1.4.

Prior to applying flow, all systems were equilibrated in the absence of external driving, $\textbf{F}^{\textrm{flow}}_i$. All reported observables were obtained either by averaging over multiple simulation runs or by averaging over time.

**Fig. 3:**
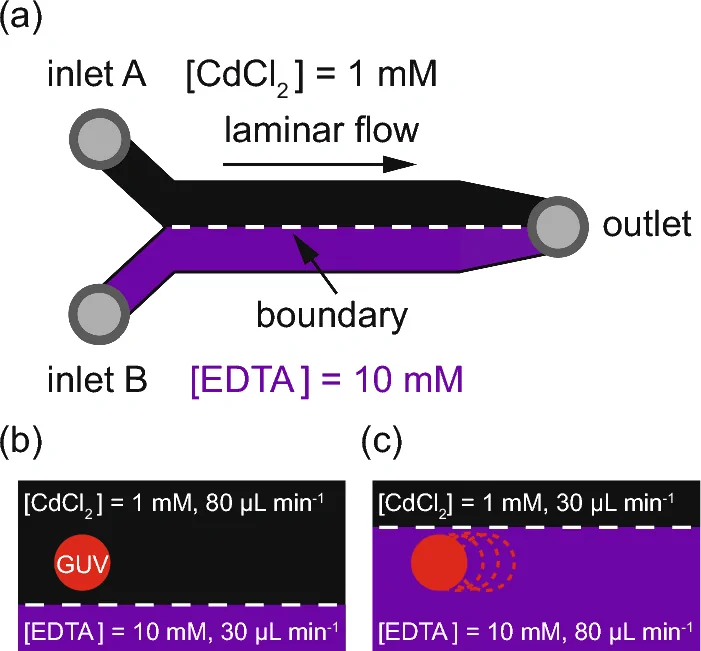
Experimental setup. **a** Y-shaped microfluidic channel with 2 × inlets allows for mixing of two laminar flows. The boundary between Flow A ([CdCl$_2$] = 1 mM) and Flow B ([EDTA] = 10 mM) is movable by varying the ratio of flow rates. **b** GUV exposed to Flow A firmly adheres to the surface. **c** Exposure to EDTA buffer weakens the adhesion, and GUV starts moving

## Results

### Microfluidic control of adhesion switching

We first establish a microfluidic system to dynamically modulate vesicle–substrate adhesion via exchange of buffer in contact with vesicles. The experimental setup consists of a Y-shaped microfluidic channel that allows two laminar streams to flow side-by-side, enabling a controlled and spatially well-defined chemical environment (Fig. 3a). One inlet supplies a buffer containing 1 mM CdCl$_2$ (Flow A) that promotes strong adhesion. The other inlet delivers a buffer containing 10 mM EDTA (Flow B), which removes Cd$^{2+}$ ions from the pAA-Cys5 brushes. The position of the boundary between two flows can be tuned by adjusting the relative flow rates, allowing a vesicle to be exposed sequentially to distinct adhesion conditions without mechanical perturbation. Fluorescence labeling of the EDTA stream confirms the stability and sharpness of the interface over millimeter distances (Figures S4 and S5), ensuring that the observed vesicle response is governed by well-controlled switching of two flows. Figure 3b exemplifies the shift of flow by changing the flow rates. When Flow A and Flow B are mixed at a ratio of 80: 30 μL min$^{-1}$, giant unilamellar vesicles (GUVs) near the channel center are exposed to CdCl$_2$-containing buffer, facilitating firm adhesion to the pAA-Cys5-coated substrate. Once the ratio between flow rate A and flow rate B is switched to 30: 80 μL min$^{-1}$, GUVs are exposed to EDTA-containing buffer that reduces adhesion, leading to partial detachment and the onset of vesicle movement. This reversible switching provides a direct experimental handle to probe the dynamic transition between adhered and mobile states under flow.

**Fig. 4:**
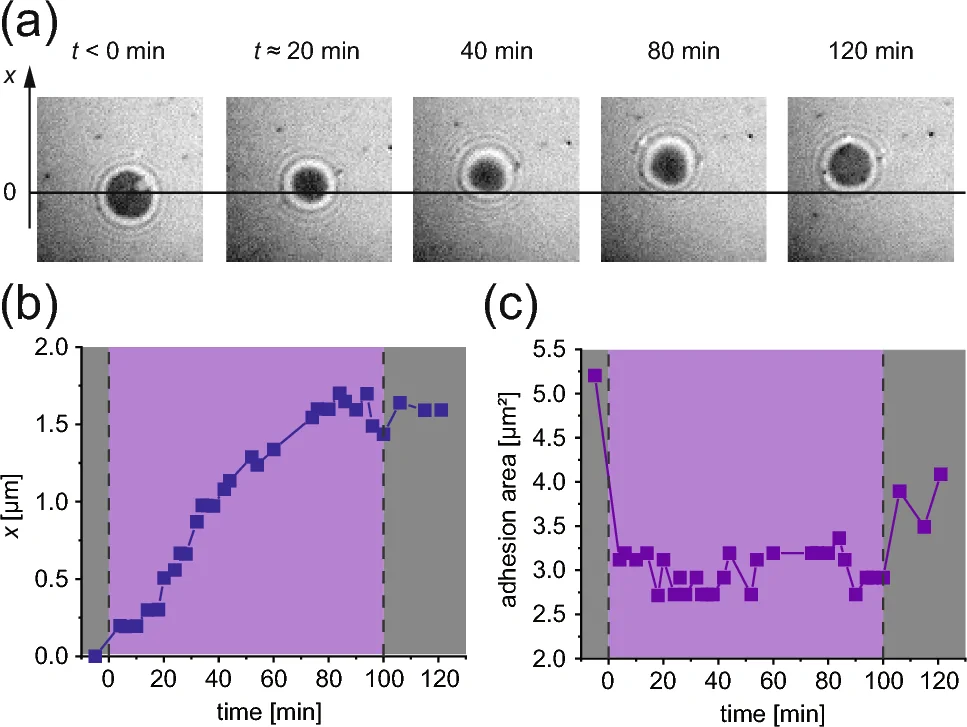
Vesicle movement observed by experiments. **a** RICM images of a GUV under flow. **b** Centroid position in the flow direction recorded over time. The gray-shaded time zone corresponds to exposure to CdCl$_2$ (Flow A), while the purple zone corresponds to exposure to EDTA (Flow B). **c** The corresponding time series of adhesion area

### Dynamic transition between wetting and non-wetting states

The vesicle dynamics under flow are quantified using RICM imaging, which allows simultaneous tracking of the position and adhesion area of GUVs. Time-resolved measurements reveal a clear correlation between adhesion strength and vesicle mobility. Figure 4a represents the RICM images taken at *t* < 0 min, 20 min, 40 min, 80 min, and 120 min. Initially (*t* < 0 min), the ratio of flow rates is adjusted as 80: 30 μL min$^{-1}$. Here, the vesicle was exposed to CdCl$_2$, and the vesicle remains immobilized despite the imposed flow corresponding to the shear stress of 0.022 Pa. After confirming that the vesicle is immobilized at the initial position (*x* = 0 μm), the ratio of flow rates is switched to 30: 80 μL min$^{-1}$ at *t* = 0 min. Thus, the vesicle is exposed to EDTA and the vesicle starts to exhibit a motion in the direction of the flow (Fig. 4b). Of note, this transition is accompanied by a pronounced decrease in adhesion area. As shown in Fig. 4c, the adhesion area drops rapidly, almost by a factor of two, after the chemical switch and remains at a reduced level while the vesicle moves. When the flow is switched back to CdCl$_2$, the vesicle motion ceases, and the adhesion area increases back to the initial level, demonstrating reversibility of the process. These observations indicate that vesicle motion is governed by a competition between hydrodynamic drag and adhesion-mediated friction. Strong adhesion leads to a large contact area and high friction, resulting in an arrested state. In contrast, reduced adhesion decreases the contact area and hence friction, allowing the vesicle to move under flow. Importantly, the rapid change in adhesion area compared to the slower center-of-mass displacement suggests a separation of timescales: The vesicle shape adapts quickly to the local chemical environment (Fig. 4c), while translational motion evolves on longer timescales (Fig. 4b).

**Fig. 5:**
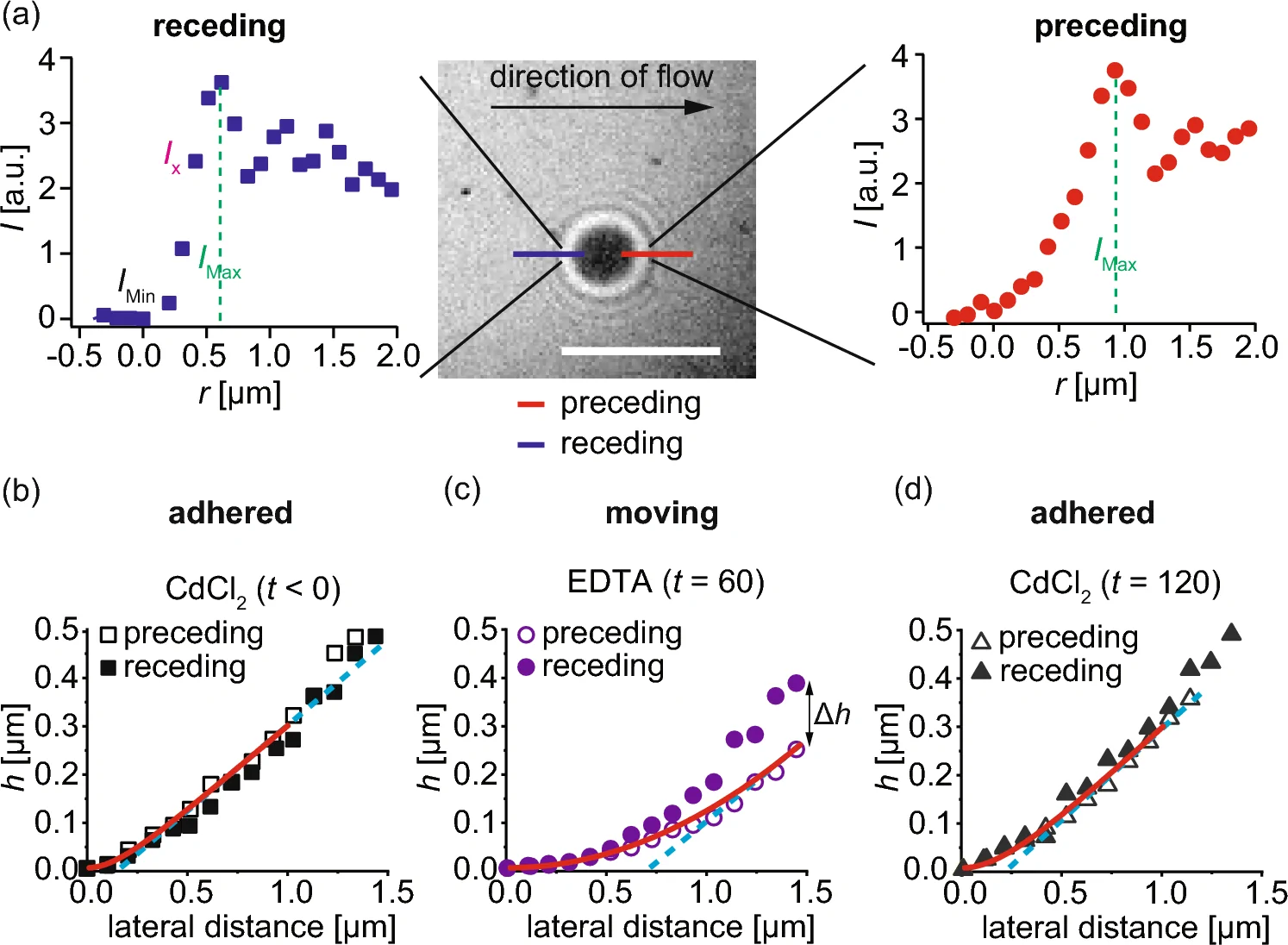
Shape analysis of GUV. **a** Intensity profiles on the preceding side (red) and receding side (blue) are plotted with intensity extrema and an arbitrary intensity ($I_\text {x}$) indicated. The normalized intensity $I_\text {norm}$ is defined in Eq. 5. The periodic ambiguity of the intensity-height relation is resolved by separating ascending and descending branches while taking the fringe sequence into account.[41, 42] For descending branches $I_\text {norm}$ = 1–$I_\text {norm}$. The height profile h($I_\text {x}$) is then calculated following Eq. 4. Height profiles of the membrane near the surface monitored at 3 time points; open symbols (preceding side); filled symbols (receding side). Flow was switched from Flow A to Flow B at *t* = 0 and from Flow B to Flow A at 100 min. **b** adhered vesicle at t< 0 (before switching, CdCl$_2$), **c** moving vesicle at *t* = 60 min (after switching, EDTA), **d** *t* = 120 min (after switching back, CdCl$_2$)

**Table 1:** Shape indices

	λ (μm)	α (°)	$C_{\text {max}}$ (μm$^{-1}$)
Adhered (t< 0 min)			
preceding	0.17	19.4	2.03
receding	0.20	21.4	1.83
Moving (*t* = 60 min)			
preceding	0.73	18.7	0.45
receding	0.56	23.4	0.73
Adhered (*t* = 120 min)			
preceding	0.23	20.6	1.54
receding	0.19	21.8	2.04

Capillary length λ, contact angle α, maximum curvature $C_{\text {max}}$

### Flow-induced shape asymmetry and dynamic deformation

Beyond translational motion, RICM imaging reveals significant changes in vesicle shape upon adhesion switching. Figure 5a represents a typical RICM image of a vesicle exposed to the flow of EDTA-containing buffer. The intensity profiles in the preceding and receding directions are asymmetric, suggesting that the vesicle deforms under flow. To quantify this deformation, we reconstruct the membrane height profile from RICM intensity using established interference analysis. This allows us to precisely resolve the vesicle shape near the substrate. As shown in Fig. 5b, a vesicle exhibits a symmetric shape even under flow when it firmly adheres to the surface in the presence of CdCl$_2$ (black symbols). In contrast, when adhesion is weakened by EDTA flow (Fig. 5c), the shape of a moving vesicle becomes asymmetric along the flow direction (purple symbols). The reconstructed profiles show that the vesicle develops a forward tilt under flow, which shares a common feature with the advancing and receding droplet of a simple Newtonian fluid sliding on a tilted substrate [1, 56]. The height profile on the preceding side is lower than that on the receding side. The origin of the asymmetric deformation can be attributed to hydrodynamic stresses acting on the vesicle. Flow exerts a shear force that tends to tilt the vesicle, while adhesion and membrane bending resist deformation. The resulting shape reflects a balance between these competing effects. This asymmetry is reversible upon restoring strong adhesion (gray symbols) as shown in Fig. 5d. To quantify these observations, the contact curvature $C_{max}$ = $\alpha /\lambda$ was determined following the previous account[18], where α is the contact angle and λ is the capillary length. In the initial state at t< 0 min, the curvature on the preceding side ($C_{\text {max, preceding}}$ = 2.03 $\mu m^{-1}$) is similar to the curvature on the receding side ($C_{\text {max, receding}}$ = 1.83 $\mu m^{-1}$). In the moving state at *t* = 60 min, the curvature on the receding side ($C_{\text {max, receding}}$ = 0.73 $\mu m^{-1}$) is around 40% higher compared to $C_{\text {max, preceding}}$ = 0.45 $\mu m^{-1}$. Switching back to the adhered state again results in similar curvatures of $C_{\text {max, receding}}$ = 2.04 $\mu m^{-1}$ and $C_{\text {max, preceding}}$ = 1.54 $\mu m^{-1}$ Table 1. These findings show that the shear stress at flow rates of 110 μL min$^{-1}$ does not cause major differences between the curvatures of the adhered states. It should be noted that it is technically challenging to realize the whole process. Namely, the vesicle that is large enough to establish stable adhesion must adhere to the position near the channel center in order to be exposed to EDTA buffer. Furthermore, the vesicle must move by being exposed to EDTA buffer flow. As most of the vesicles either left the surface or got immobilized/pinned, we could capture the detailed vesicle shape throughout the whole process only once. On the other hand, increasing the shear stress on vesicles in the adhered state to 0.039 Pa results in distinctly higher curvatures on the receding side compared to the preceding side (see SI, Figure S6). This pronounced asymmetry in terms of vesicle shape compared to the one presented in Fig. 5 can be explained by the large difference in the capillary number (Eq. 3), which relates viscous stress to the membrane-bending force. Doubling the vesicle radius from 4 to 8 μm and increasing the shear stress from 0.022 to 0.039 Pa results in an increase in the capillary number, Ca, from around 14 to 200. This suggests that the transition from the bending-dominated regime to the one dominated by viscous stress starts at Ca >14.

### Static adhesion states and curvature analysis

To provide a reference for the dynamic experiments, we characterize vesicle shapes under static (no-flow) conditions at different CdCl$_2$ concentrations (Figure S7). At low CdCl$_2$ concentration (0.25 mM), vesicles exhibit weak adhesion with a relatively small contact area and shallow curvature near the substrate (Figure S7a). At higher concentration (1 mM), adhesion is significantly stronger, leading to a larger contact area and increased curvature near the rim (Figure S7b). Quantitative analysis of the height profiles reveals an increase in the maximal curvature C$_{max}$ with increasing adhesion strength. This trend is consistent with theoretical predictions based on the balance between adhesion energy and membrane-bending rigidity (Eq. 2). Stronger adhesion leads to a sharper rim and a flatter adhered disk. These static measurements provide a baseline for interpreting the dynamic experiments: The adhesion switching induced by Cd$^{2+}$/EDTA effectively drives the system between two distinct regimes characterized by different curvature and contact area.

### Switchable vesicle adhesion in flow: simulation

**Fig. 6:**
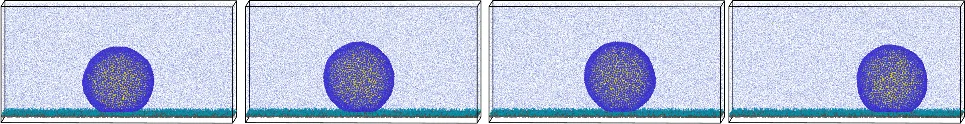
Adhesion-controlled vesicle motion under flow. Initially adhered and stationary, the vesicle begins to move when adhesion strength is reduced, then re-adheres and slows significantly when adhesion is increased (Visualized using OVITO [57])

In the experiment, vesicles adhering to switchable substrates exhibit distinct dynamical regimes under shear flow, depending on the adhesion strength. At strong adhesion, the vesicle remains essentially immobilized despite the applied flow. As the adhesion strength decreases, the vesicle translates across the surface. Re-increasing the adhesion enlarges the contact area and deformation, arresting the motion again. In the following, we first compare the dynamical changes in the vesicle shape and position in our simulations with the experimental findings (Fig. 6). We then analyze the corresponding contact area and contact curvatures. To gain deeper insights into the underlying mechanisms, we further investigate the nonequilibrium steady shape of vesicles under constant shear flow for different capillary numbers, Ca.

**Fig. 7:**
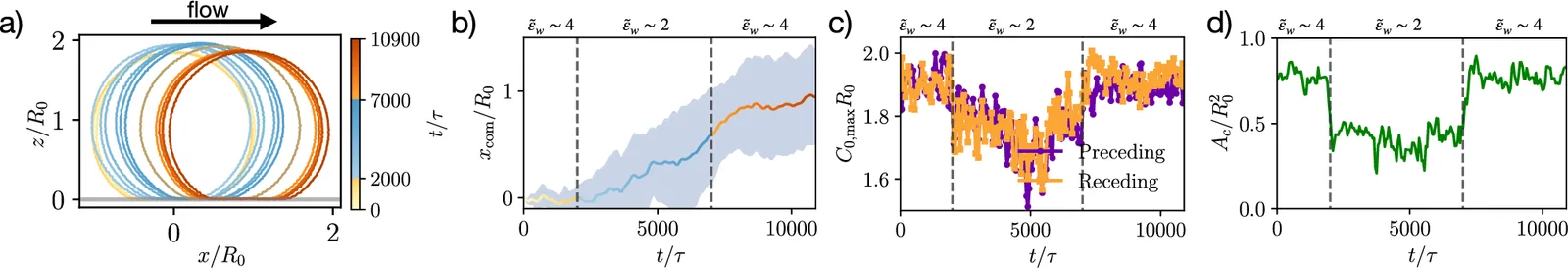
Dynamics of adhered vesicles under time-varying adhesion strength. **a** Central cross sections parallel to the flow direction show a forward tilt and a vesicle height increase when adhesion decreases from $\tilde{\varepsilon }_w \approx 4$ (orange/red) to $\tilde{\varepsilon }_w \approx 2$ (blue). Color indicates time. **b** Center-of-mass displacement shows arrested motion at strong adhesion ($\tilde{\varepsilon }_w \approx 4$), translational motion at reduced adhesion ($\tilde{\varepsilon }_w \approx 2$), and re-arrest upon returning to strong adhesion. The vesicle moves at constant velocity $v_x \approx 0.001 \ r_c^\textrm{DPD}/\tau$ during the low-adhesion phase. The shaded area indicates the standard deviation of the displacement across the four evaluated runs. **c** Preceding (purple) and receding (orange) contact curvatures quantify the flow-induced asymmetry. Both curvatures are higher at $\tilde{\varepsilon }_w \approx 4$ than at $\tilde{\varepsilon }_w \approx 2$, consistent with stronger adhesion. **d** Contact area $A_c$ responds quickly to adhesion switching (dashed lines), decreasing from $A_c/R_0^2 \approx 0.75$ to $A_c/R_0^2 \approx 0.4$ when adhesion is reduced, then recovering to its initial value when adhesion is restored

Previous investigations [18] indicated that the experimentally observed vesicles approximately correspond to dimensionless adhesion strengths of $\tilde{\varepsilon }_w = \Delta \gamma _w R_0^2/\kappa \approx 4$ and $\tilde{\varepsilon }_w \approx 2$. To reproduce dynamics comparable to those observed experimentally, we applied a small driving force, $F_0 = 0.00001 \ k_{B} T / r_c^\textrm{DPD}$. In addition to varying the adhesion strength, we switched the activity of the wall springs between the membrane and the substrate from $z_\textrm{wlsp}^\textrm{ves } (\tilde{\varepsilon }_w = 4) = 8$ to $z_\textrm{wlsp}^\textrm{ves } (\tilde{\varepsilon }_w = 2) = 4$. This choice is physically plausible because stronger adhesion is also expected to slow the attachment–detachment dynamics at the rim of the contact zone. Note that (i) increasing the wall-spring activity does not alter the equilibrium structure, and (ii) substrate friction generates an effective no-slip boundary condition (*vide infra* subsection 2.3.4 and Figure S3a). We then observed the vesicle shape and tracked the center-of-mass (COM) movement (see Fig. 7 panels a and b, respectively), averaging over four trajectories.

Analysis of the experimental RICM images revealed asymmetric vesicle shapes under flow. Figure 7a shows the central cross section parallel to the flow direction for successive times. The colorbar indicates time, with orange/red tones for $\tilde{\varepsilon }_w \approx 4$ and blue tones for $\tilde{\varepsilon }_w \approx 2$. As adhesion is decreased, the vesicle height increases while the contact area decreases. As adhesion increases again, the contact area increases, and the height decreases. The vesicle is tilted forward in the flow direction, creating an asymmetry between the preceding and receding contact lines.

At $\tilde{\varepsilon }_w \approx 4$, the motion of the vesicle’s center of mass in flow direction is negligible over the simulation duration (see panel b). Reducing the adhesion to $\tilde{\varepsilon }_w \approx 2$ (changes in adhesion indicated by dashed vertical lines) triggers translational motion with an average velocity of $\langle v_x \rangle \approx 0.001 \ r_c^\textrm{DPD}/\tau$. Restoring the adhesion to $\tilde{\varepsilon }_w \approx 4$ significantly reduces the motion after a total displacement of $\Delta x \approx 0.9 R_0$.

The center-of-mass displacement along the flow direction increases on average linearly between the two dashed lines, consistent with a mean drift velocity imposed by the driving force. The apparent ruggedness reflects the influence of thermal fluctuations on this drift—the vesicle undergoes a biased random walk rather than directed stick–slip motion as documented in other studies, e.g., Refs. [58–60]. This is additionally supported by data in Figure S3.

The delay of velocity reduction observed upon increasing adhesion strength arises as the vesicle shape —quantified, e.g., by the contact area and the pre- and receding contact curvatures—relaxes on a timescale of $\tau _\textrm{shape} \approx 75 \tau$. Although the adhesion strength, as well as the wall-spring density, adapt quasi-instantaneously upon switching, the significant slowing of translational motion requires the shape of the vesicle, and in particular the area and geometry of the rim of the contact zone, to (re)adjust. This is because resistance to translational motion arises from the kinetics of wall-spring rupture at the receding contact edge. A useful measure of the resistance to translational motion is the number of wall springs that must rupture per unit displacement of the vesicle, $N_\textrm{wlsp}^\mathrm{ves-wall}|_\mathrm{receding \ edge} \propto z_\textrm{wlsp}^\textrm{ves} l_\textrm{rc} \propto z_\textrm{wlsp}^\textrm{ves}\sqrt{A_c}$, where $l_\textrm{rc} \sim \sqrt{A_c}$ is the arc length at the receding edge. Both $A_c$ and $z_\textrm{wlsp}^\textrm{ves}$ and thereby $N_\textrm{wlsp}^\mathrm{ves-wall}|_\mathrm{receding \ edge}$ increase with adhesion strength. Consequently, the number of rupture events required for forward motion also increases, rendering the rate of simultaneous rupture of a sufficient number of wall springs progressively less probable. This reduces the vesicle velocity.^2^

While this delayed velocity reduction is visible in our simulations, it is not directly observable in experiments due to differences between the shape-relaxation time and sampling rates. For $\kappa =25k_BT$, $R_0=8\mu$m, and $\eta =10^{-3}$Pa s, we obtain for the shape-relaxation time, $\tau _\textrm{shape} \sim \eta R_0^3/\kappa \approx 5$ s. Nevertheless, the underlying physical mechanism and the associated changes in vesicle shape remain comparable between simulation and experiment.

To further quantify the vesicle dynamics, we investigate the vesicle’s contact area with the substrate, defined as

11 $$\begin{aligned} A_c = \pi r_c^2, \quad r_c = \max \{r_i : z_i < z_\textrm{threshold}\}, \end{aligned}$$

with $z_\textrm{threshold}=0.02r_c^\textrm{DPD}$, and the pre- and receding contact curvatures, which are presented in Fig. 7c. Initially, we observe $A_c/R_0^2 \approx 0.75$ at strong adhesion. Upon reducing adhesion, $A_c$ rapidly decreases to $A_c/R_0^2 \approx 0.4$, remaining stationary during translational motion. When adhesion is increased again, $A_c$ returns to its initial value over the timescale of $\tau _\textrm{shape} \approx 75 \tau$.

The correlation between contact area and mobility suggests a direct connection between adhesion strength and vesicle dynamics. Since the vesicle is tank-treading, the slip velocity of the membrane in the contact area is essentially zero (see Figure S3a), and there is no sliding friction. Instead, vesicle motion is governed by the viscous coupling between the vesicle membrane and the surrounding solvent, and by the dynamics of the wall-spring de-/attachment at the edge of the contact zone.

To quantify the asymmetry of the vesicle in motion, observed both in experiment and in simulation, we define the preceding and receding contact curvatures as

12 $$\begin{aligned} C_\textrm{pre} = \max \{C : x< x_\textrm{com}, z < z_\textrm{threshold}\}, \end{aligned}$$

13 $$\begin{aligned} C_\textrm{re} = \max \{C : x > x_\textrm{com}, z < z_\textrm{threshold}\}, \end{aligned}$$

where the maximum is taken at the respective half of the contour. At $\tilde{\varepsilon }_w \approx 4$, both curvatures adopt higher average values ($C_\textrm{pre}R_0 \approx C_\textrm{re}R_0 \approx 1.9$) than at $\tilde{\varepsilon }_w \approx 2$ ($C_\textrm{pre}R_0 \approx C_\textrm{re}R_0 \approx 1.75$), consistent with the stronger adhesion producing increased contact angles.

Visual inspection of the vesicle shapes (Fig. 7a) reveals a slight forward tilt. Under flow, hydrodynamic stresses generate a torque that inclines the vesicle, reducing the curvature at the leading edge, where the flow pushes the membrane toward the wall, and increasing it at the trailing edge, where the membrane is pulled away from the wall. However, these curvature differences remain comparable to the statistical fluctuations arising from the finite simulation time. Moreover, the curvatures do not attain a clear plateau during the interval of weakened adhesion, and the center-of-mass motion remains irregular. We therefore conclude that the vesicle does not fully equilibrate into a steady moving state within this regime.

A similar front-rear asymmetry has been observed in capsules under shear flow [61], where the tilt angle depends on the balance between viscous and elastic forces, which is quantified by the capillary number, Ca (see Eq. 3).

### Deformation dependence on flow speed for Ca ≪1

**Fig. 8:**
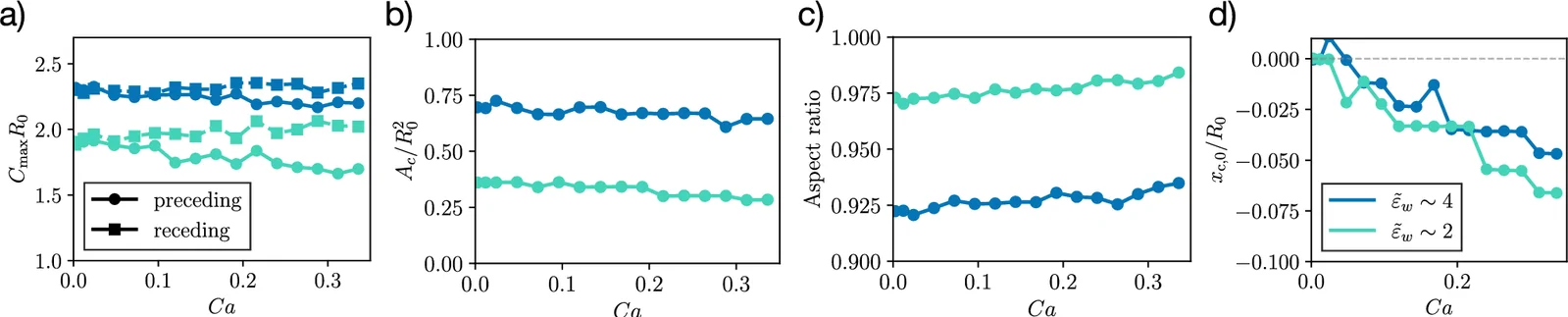
Flow-induced changes in vesicle geometry at Ca ≪1. **a** Preceding (spheres) and receding (squares) contact curvatures as functions of flow force. The curvatures separate with increasing $F_0$ and, in turn, increased *Ca*, reflecting increased asymmetry. This divergence occurs at lower flow forces for the weakly adhered vesicle (light blue) compared to the more strongly adhered vesicle (dark blue). **b** Contact area $A_c/R_0^2$ remains approximately constant across the flow range for both adhesion strengths, indicating that the contact footprint size is primarily controlled by adhesion rather than flow. **c** Aspect ratio (height/width) increases minimally with flow. **d** The position of the contact area center relative to the center of mass shifts opposite to the flow direction as the vesicle tilts

To robustly quantify curvature asymmetry and link it to hydrodynamic forces acting on the adhered vesicle, better statistics on the vesicle shapes are necessary. This section investigates the nonequilibrium steady-state shapes of vesicles adhered to substrates under constant flow and adhesion strength, thereby enabling a more precise characterization of this effect.

An adhered vesicle in constant flow adapts a nonequilibrium steady shape determined by the balance between adhesion, membrane bending, and hydrodynamic stress. We now examine how flow strength modulates this nonequilibrium steady vesicle shape, by progressively increasing the flow force $F_0$ applied to vesicles at $\tilde{\varepsilon }_w \approx 4$, $z_\textrm{wlsp}^\textrm{ves}=8$ and $\tilde{\varepsilon }_w \approx 2$, $z_\textrm{wlsp}^\textrm{ves}=4$.

**Fig. 9:**
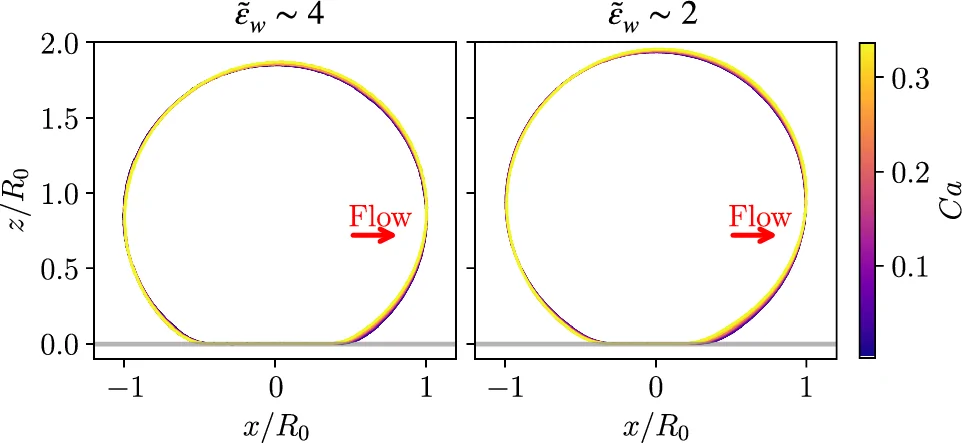
Nonequilibrium steady vesicle shapes for increasing flow force at two adhesion strengths. Cross sections show the central slice parallel to the flow direction, colored by local flow speed. Stronger adhesion ($\tilde{\varepsilon }_w \approx 4$, left): Vesicle exhibits minimal tilt even at high flow forces. Weaker adhesion ($\tilde{\varepsilon }_w \approx 2$, right): Vesicle shows a slightly more pronounced forward tilt with increasing flow speed. The geometric observables shown in Fig. 8 are extracted from these shapes

Figure 9 shows the central vesicle cross section in flow direction, colored by flow speed. In both settings, the vesicle tilts slightly as flow speed increases, with the effect being more pronounced for the weakly adhered vesicle. This tilt is quantified by the offset between the center of mass and the center of the contact area (Fig. 8d), which increases progressively with $F_0$. This stronger tilt occurs as the vesicle extends higher above the wall, exposing more surface area to the faster-moving fluid in the upper part of the shear flow.

To quantify these trends, we measure four geometric observables as functions of flow force (Fig. 8): contact curvatures (panel a), contact area (panel b), aspect ratio between height and width (panel c), and the position of the center of the contact area with respect to the center of mass (panel d). The preceding and receding contact curvatures (panel a) are equal in the absence of flow, and remain approximately equal at low flow force. For the weakly adhered vesicle (light blue), the curvatures start to differ for Capillary numbers Ca >0.05. The receding curvature (squares) increases while the preceding curvature (spheres) decreases, consistent with the forward tilt observed in Fig. 9. This asymmetry arises from the nonuniform distribution of hydrodynamic forces across the vesicle membrane. Flow exerts stronger drag on the upper, more exposed portions of the vesicle, creating a net horizontal force that pushes the vesicle forward and tilts it in the flow direction. For the stronger adhered vesicle (dark blue), the curvatures remain relatively close even at higher hydrodynamic forces. The reduced height also reduces exposure to the regions of high shear, and stronger adhesion resists flow-induced deformation. Similar suppression of flow effects by strong adhesion has been observed in vesicle membrane flow studies  [52].

The contact area $A_c$ (panel b of Fig. 8) remains approximately constant, only decaying slightly across the examined flow range for both adhesion strengths. Similarly, the aspect ratio (panel c) increases only by about 1%. This indicates that the contact area size and the vesicle shape are primarily determined by the adhesion-bending equilibrium rather than by hydrodynamic forces in the current parameter range, Ca ≪1. The effect of shear is most visible in the shift of the position of the contact area center relative to the center of mass (Fig. 8d), which progressively shifts opposite to the flow direction as $F_0$ increases and quantifies the tilt observed in Fig. 9. At $\tilde{\varepsilon }_w \approx 2$, the shift is more pronounced, reaching $\Delta x/R_0 \approx -0.07$ at the highest flow rate, while the strongly adhered vesicle at $\tilde{\varepsilon }_w \approx 4$ shows smaller displacements.

### Fast flow: lift force vs buoyancy

**Fig. 10:**
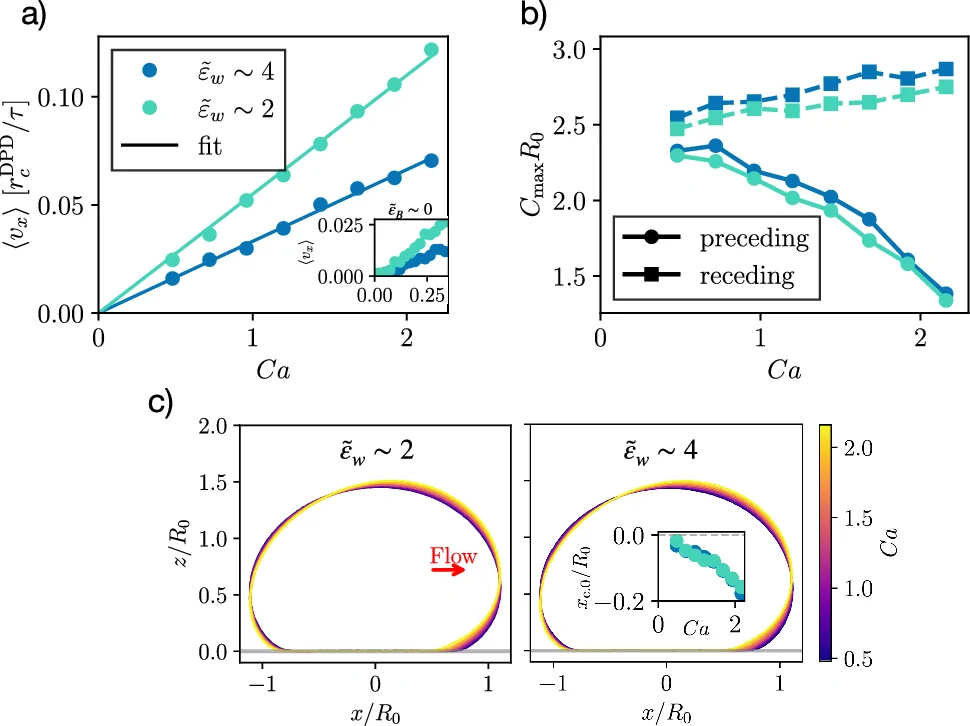
Center-of-mass motion and quasi-static shape of vesicles in strong flow with added buoyancy. **a** The steady-state *x*-direction velocity of the vesicle as a function of Ca. **b** Preceding (circles) and receding (squares) contact curvatures differ strongly under high shear. The preceding curvature decreases more than the receding curvature increases, reflecting asymmetric membrane shape. The difference between adhesion strengths is more pronounced at the preceding edge than at the receding edge. **c** Cross sections for $\tilde{\varepsilon }_w \approx 2$ and $\tilde{\varepsilon }_w \approx 4$ at increasing flow forces show pronounced forward tilt and flattening. As the shapes are dominated by the balance of buoyancy, shear stress, and volume conservation, with adhesion playing a minor role, they look almost identical. Inset presents offset between center-of-mass and center of contact area for both $\tilde{\varepsilon }_w \approx 2$ (light blue) and $\tilde{\varepsilon }_w \approx 4$ (dark blue)

Vesicles in shear flow experience an upward lift force [54, 62, 63] that scales as [63]

14 $$\begin{aligned} F_l \propto \eta \dot{\gamma }R_0^2 \Gamma , \end{aligned}$$

where Γ is a dimensionless function depending on the vesicle geometry and distance to the substrate. This lift force arises from the nonuniform flow field around the tilted vesicle [64]. Under shear, the vesicle adopts a forward-tilted configuration while tank-treading. This tilt breaks the symmetry of the flow between the preceding and receding edges, creating an asymmetric pressure distribution: The flow accelerates over the inclined upper surface at the preceding edge (reducing pressure) and decelerates beneath at the receding edge (increasing pressure). The resulting pressure imbalance integrates to a net upward force. At low Reynolds number, where inertia is negligible, this is a purely viscous effect arising from the geometry-induced stress asymmetry [63]. Spherical particles experience no such lift due to flow reversibility, emphasizing that shape deformation and tilt are essential for lift generation. At sufficiently high shear rates, this lift force overcomes adhesion, causing detachment, a phenomenon observed in both experiments and simulations [53, 54, 63, 65].

In the previous sections, the shear rate was sufficiently low to prevent detachment. To study stronger flow-induced deformations while maintaining adhesion, we introduce downward buoyancy by applying $F_\textrm{buoy} = -0.05 \ k_\textrm{B}T / r_c^\textrm{DPD}$ to each solvent particle inside the vesicle, mimicking a density mismatch between the vesicle interior and exterior. The buoyant force, $F_\textrm{buoy} = \Delta \rho V g \propto R_0^3$, scales differently than the lift force with the vesicle size, making larger vesicles slightly less prone to detach. Here, we applied a downward buoyancy of $\tilde{\varepsilon }_B = \frac{\Delta \rho g R_0^4}{\kappa } = -35$ to keep the vesicle stuck to the substrate. With the introduced buoyancy, we studied capillary numbers up to Ca≈2.2.

The vesicle shape in this regime results from the interplay of bending energy, buoyancy, membrane–volume conservation, and hydrodynamic stresses under flow, with the adhesion potential playing a comparatively minor role in determining the overall geometry. As the buoyancy force pushes the vesicle toward the wall, the enclosed volume cannot change, so the contact area expands and the membrane is placed under tension. The resulting shape resembles a more strongly adhered vesicle, but the flattening is primarily driven by buoyancy and volume conservation rather than by the adhesion strength. The deformed equilibrium shape is characterized by a reduced volume of $\nu = V/V_0 \approx 0.93$.

Figure 10a shows the steady-state center-of-mass velocity $\langle v_x \rangle$ as a function of Ca. At Ca=0, the vesicle is adhered and stationary. We observe a linear increase in the vesicle velocity with Ca, consistent with a shear-dominated vesicle response. The vesicle is tank-treading, as seen by tracking the contact-zone vertex velocities.

We furthermore observe a consistently higher velocity for the $\tilde{\varepsilon }_w \approx 2$ vesicle, with a dependence on Ca of $\langle v_x \rangle \tau / r_c^{DPD}=0.055$Ca in comparison to $\langle v_x \rangle \tau / r_c^{DPD}=0.033$Ca for the $\tilde{\varepsilon }_w \approx 4$ vesicle. As the wall spring density is much higher for $\tilde{\varepsilon }_w \approx 4$, this is to be expected. The inset provides the steady-state center-of-mass velocity $\langle v_x \rangle$ as a function of Ca for the simulations carried out at Ca ≪1 without buoyancy ($\tilde{\varepsilon }_B = 0$). To investigate this further, we analyze the vesicle shapes in more detail.

Figure 10c shows vesicle cross sections as well as the forward tilt of the vesicles under these stronger flow conditions. The added buoyancy significantly flattens the vesicle shape, reducing its height and thus its exposure to regions of higher flow speed. Despite this flattening, the forward tilt becomes more pronounced due to the stronger flow, which is emphasized by the inset showing the offset between the center of mass and the center of the contact area. Despite the buoyancy pushing the vesicle toward the wall, the contact area decreases with increasing Ca (see Fig. 10c). With the decrease in the contact area, the velocity of the vesicle increases as the amount of wall springs needing to rupture per unit displacement scales with $\sim \sqrt{A_c}$. This also leads to a slightly higher velocity for $\tilde{\varepsilon }_w=2$, as this vesicle has a slightly smaller contact area due to the weaker adhesion potential. Nonetheless, as the shapes for both adhesion strengths are very similar, the major difference in determining the velocity difference is the areal wall spring density.

The preceding and receding curvatures change drastically under these conditions, as expected (see Fig. 10b). Interestingly, the preceding curvature decreases much more than the receding curvature increases. This asymmetric response reflects different effects at the front and rear of the vesicle. At the preceding edge, the membrane is pushed toward the substrate by the flow, causing the contact zone to advance and the contact edge to become flatter, reducing the local curvature. At the receding edge, the membrane is dragged forward and away from the substrate, causing the contact area to recede as the membrane peels off, increasing the contact curvature. At both edges, the dynamics are governed by the wall-spring dynamics. At the front, new springs attach as the contact advances, while the springs at the receding edge must rupture before the membrane can lift off.

The contact curvature $C_\textrm{max}($Ca=0) in the absence of flow is given by the balance of adhesion and bending. Any deviation $\Delta C = |C_{\max }-C_\textrm{max}($Ca=0)| costs energy, $\Delta E/A_\textrm{edge} \sim \alpha \kappa (\Delta C)^2$, quadratic in the change in curvature, where α is a geometric prefactor of order unity. In the linear-response regime, Ca $\lesssim \mathcal{O}(1)$, we expect the change of bending stress in response to a curvature change, $\Delta C = |C_{\max }-C_\textrm{max}($Ca=0)|, be proportional to the viscous shear stress, $\frac{\kappa \Delta C}{R_0^2} \sim \eta \dot{\gamma }$. This indicates linear scaling, $\Delta C R_0 \sim$ Ca, consistent with Fig. 10b. The slopes of this linear relation, however, differ between the two edges and between adhesion strengths.

These steady-state shape measurements under constant flow and adhesion provide the context for interpreting the dynamic adhesion-switching experiments of the previous section. During the adhesion-switch cycle in the simulation, the vesicle did not reach the fully steady shapes characterized here. The delayed reduction in velocity upon increasing adhesion strength reflects the time required for equilibration. The curvature asymmetries and tilts observed here under steady flow represent the target configurations toward which the vesicle relaxes while moving and match the deformations observed in the experiment.

## Discussion and conclusion

Combining the dynamic and static observations, a consistent physical picture emerges. The key control parameters governing vesicle behavior are the effective adhesion strength, $\tilde{\varepsilon }_w$, which determines the vesicle shape—including both the adhesion area and the maximum membrane curvature near the substrate—and the capillary number, Ca.

Across the entire parameter range studied, vesicle motion proceeds exclusively via tank-treading. Consequently, the membrane does not slip past the substrate. The angular tank-treading velocity and the translational velocity are therefore directly related, and there is no friction between the membrane and the substrate. Dissipation instead originates from two primary mechanisms: (i) the relative motion between the vesicle and the surrounding viscous solvent, together with the viscous dissipation occurring within the solvent itself (present even without the vesicle), and (ii) the dynamics of attaching and detaching at the rim of the contact zone. The former hydrodynamic friction depends on the vesicle shape, which is itself modulated by the interplay of adhesion, bending elasticity, and flow. The latter is controlled by the areal density and kinetics of wall springs. The tank-treading velocity at fixed vesicle shape scales linearly with Ca. However, increasing the contact area and wall-spring density by increasing adhesion strength suppresses tank-treading and thereby strongly reduces translational motion on the observed time scale. By contrast, in the weak-adhesion regime, shear flow drives faster vesicle transport.

The deviation of the contact curvature from its zero-flow value follows a linear scaling with Ca for both the preceding and receding edges across the range of studied capillary numbers 0< Ca<2.2. This behavior arises from the perturbation of the local bending–adhesion balance at the contact edge by viscous stresses.

Simultaneously, shear flow induces a characteristic shape asymmetry governed by the interplay of hydrodynamic stresses, bending elasticity, and adhesion. This asymmetry becomes more pronounced at lower adhesion strengths. In both experiments and simulations, the preceding edge curvature decreases while the receding edge curvature increases. The resulting forward tilt resembles the asymmetry between advancing and receding contact lines familiar from moving droplets on solid substrates. As asymmetric vesicle shape could result from unique flow profiles inside the vesicle, future studies of hydrodynamic profiling of fluid inside the vesicles by the combination of fluorescently labeled nanoparticles and spinning disk confocal microscopy could be highly interesting [66].

Shear flow also generates a hydrodynamic lift force [54, 62, 63], cf. Eq. 14, that progressively deforms the vesicle shape and can ultimately induce desorption [53, 54, 63, 65]. To access the regime Ca >1, we employ buoyancy to counteract the shear-induced lift force. Since the buoyancy force $F_b \propto R_0^3$ grows faster with vesicle size than the lift force $F_l \propto R_0^2 \Gamma$, larger vesicles are comparatively more resistant to lift-off, regardless of adhesion strength.^3^

These simulation results are in qualitative accord with experiments that demonstrate that vesicle motion on substrates coated with stimulus-responsive polymer brushes can be reversibly controlled by chemical stimuli, providing a tunable platform for studying adhesion dynamics under flow. The ability to switch between states of different mobility offers a route to control vesicle transport in microfluidic devices.

## Supplementary Information

Below is the link to the electronic supplementary material.

Supplementary file 1 (mov 205531 KB)

Supplementary file 2 (pdf 43461 KB)

## Data Availability

Further details on the simulation model, all simulation parameters, and additional experimental data are available in the supplement. The simulation data are available from the authors upon reasonable request. The analysis scripts and run scripts, with initial configurations, are available under https://doi.org/10.5281/zenodo.21353361.

## Footnotes

1 While the enclosed volume remains approximately constant, the membrane area exhibits small fluctuations to accommodate shape deformations. An exemplary trajectory is shown in Figure S1.

2 Additionally, a lower vesicle velocity reduces the hydrodynamic lift force, allowing the vesicle to approach the substrate more closely and thereby further increasing the wall-spring density.

3 This holds at a fixed vesicle shape. As the shape asymmetry is itself, however, induced by the shear, there can be further dependencies hidden within Γ.
