# Supplementary material for: Vesicle adhesion in flow
Source: Eur Phys J E Soft Matter. 2026 Sep 22;49(10):87. doi: 10.1140/epje/s10189-026-00628-1 (PMC13597639; doi:10.1140/epje/s10189-026-00628-1)
Supplement: Supplementary file 1 [file 10189_2026_628_MOESM2_ESM.pdf]

Supplementary Information: Vesicle Adhesion in Flow

Lucia Wesenberg[2†], Felix Weissenfeld[1†], Kai-Uwe Hollborn[2], Motomu Tanaka[1,3*], Marcus Müller[2*]

[1*]Physical Chemistry of Biosystems, Institute of Physical Chemistry, Heidelberg University, Heidelberg, 69120, Germany.
[2]Institut für Theoretische Physik, Georg-August-Universität Göttingen, Friedrich-Hund-Platz 1, 37073 Göttingen, Germany.
[3]Center for Integrative Medicine and Physics, Graduate School of Medicine, Kyoto University, Kyoto, 606-8502, Japan.

*Corresponding author(s). E-mail(s): tanaka@uni-heidelberg.de; mmueller@theorie.physik.uni-goettingen.de;
Contributing authors: lucia.wesenberg@theorie.physik.uni-goettingen.de; felix.weissenfeld@uni-heidelberg.de;
†These authors contributed equally to this work.

S1 Model and simulation technique

S1.1 Membrane model

The vesicle membrane is modeled as a fluid, triangulated surface consisting of N_{vert} vertices connected by a dynamically rearranging network of bonds. Its bending energy is given by the discretized Helfrich Hamiltonian [1],

$$\mathcal{H}_{\text{bend}} = \frac{\kappa}{2} \sum_i (C_i - C_0)^2 A_i, \tag{S1}$$

where κ is the bending rigidity, C_i denotes the local mean curvature at vertex i, C_0 is the spontaneous curvature (set to zero in this work), and A_i is the associated vertex area.

The vertex areas are computed using the Voronoi construction for non-obtuse triangles,

$$A_{\text{Voronoi}} = \frac{1}{8} \sum_{j(i)} (\cot \alpha_{ij} + \cot \beta_{ij}) \|\mathbf{x}_i - \mathbf{x}_j\|^2, \tag{S2}$$

where α_{ij} and β_{ij} are the angles opposite to the edge (i, j). For obtuse triangles, the Voronoi area is corrected according to Ref. [2],

$$A_i = \begin{cases} A_{\text{Voronoi}} & \text{non-obtuse triangle,} \\ A_T/2 & \text{obtuse angle at vertex } i, \\ A_T/4 & \text{obtuse angle at a neighboring vertex,} \end{cases} \tag{S3}$$

with A_T the triangle area. Further details of the discretized curvature operator can be found in Refs. [2, 3].

To ensure numerical stability and prevent unphysical bond stretching or collapse, neighboring vertices interact via an attractive–repulsive

bond potential, $U_{\text{bond}} = U_{\text{att}} + U_{\text{rep}}$ [4–6],

$$U_{\text{att}}(r) = \begin{cases} k_b \dfrac{\exp[1/(l_{c0} - r)]}{l_{\max} - r}, & r > l_{c0}, \\ 0, & r \leq l_{c0}, \end{cases} \quad \text{(S4)}$$

$$U_{\text{rep}}(r) = \begin{cases} k_b \dfrac{\exp[1/(r - l_{c1})]}{r - l_{\min}}, & r < l_{c1}, \\ 0, & r \geq l_{c1}. \end{cases} \quad \text{(S5)}$$

This bond potential also effectively constrains the total membrane area.

Membrane fluidity is ensured by allowing bond-flip moves implemented via a Monte Carlo scheme. At fixed intervals, the molecular dynamics integration is paused, and randomly selected bonds are proposed to flip, connecting the previously unconnected vertices of the two adjacent triangles. The move is accepted with probability

$$p_{\text{acc}} = \min\left[e^{-(\Delta E_{\text{bend}} + \Delta U_{\text{bond}})/k_B T}, 1\right]. \quad \text{(S6)}$$

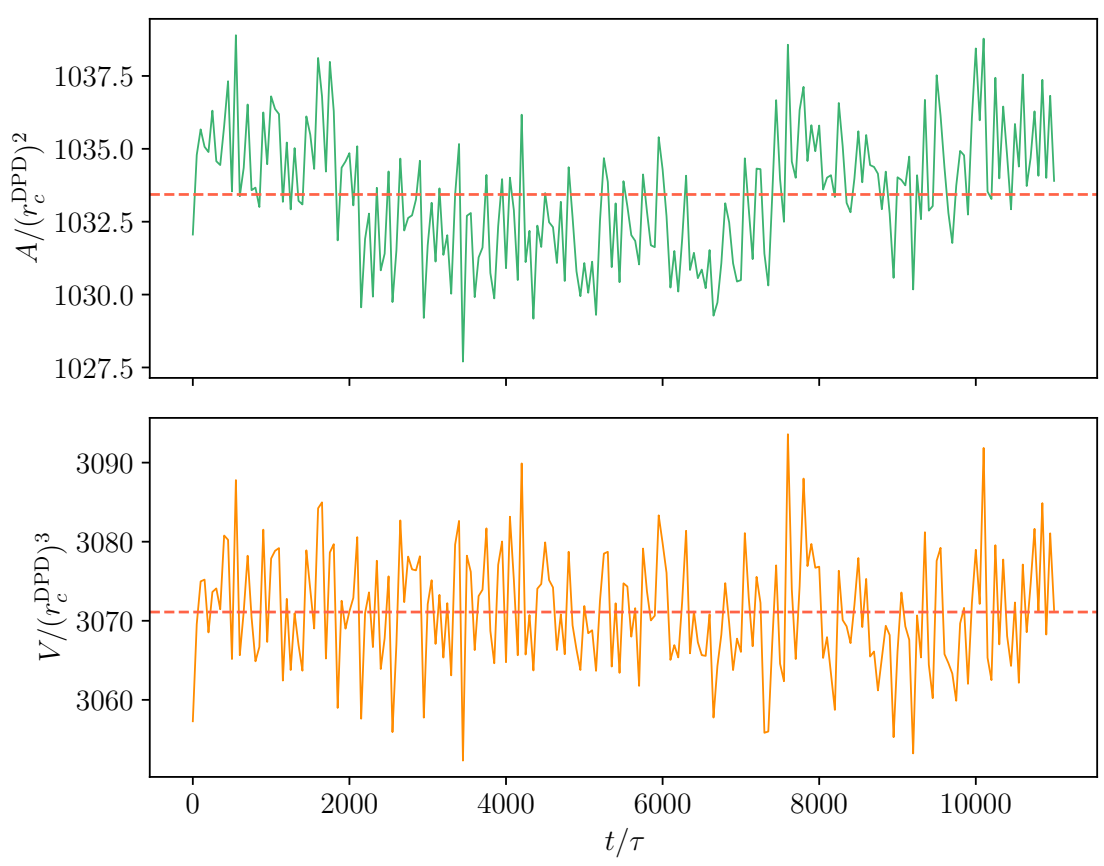

Fig. S1: Vesicle surface area and volume in units of r_c^{DPD} for an adhesion cycle where the adhesion strength is switched from $\tilde{\varepsilon}_w = 4$ to $\tilde{\varepsilon}_w = 2$ and back. The volume remains virtually constant, whereas the area slightly adapts due to the elasticity of the membrane bonds.

During the adhesion cycle, where the adhesion strength is switched from $\tilde{\varepsilon}_w \approx 4$ to $\tilde{\varepsilon}_w \approx 2$ and back, we observe that the membrane area, A, slightly increases with adhesion strength by about 0.6 ‰, whereas the volume remains virtually constant.

S1.2 Wall interactions and wall springs

The vesicle interacts with a planar wall located at $z = z_{\text{wall}}$ through a combination of conservative and dissipative interactions. Adhesion is modeled via a Mie-type wall potential acting on each membrane vertex.

Membrane-substrate friction is represented by a wall-spring thermostat. Wall springs dynamically form and break between membrane vertices/-solvent particles and immobile anchoring points on the substrate [7]. Each wall spring is described by a FENE potential,

$$U_{\text{FENE}}(r) = -\frac{1}{2} k_{\text{sp}} r_{\max}^2 \ln\left(1 - \frac{r^2}{r_{\max}^2}\right), \quad \text{(S7)}$$

where k_{sp} is the spring constant, r is the distance between the membrane vertex and wall particle, and $r_{\max}$ is the maximum extension.

In the grandcanonical ensemble, the activity, $z_{\text{wlsp}}^{\text{ves/sol}}$, controls the average number of wall springs for each wall spring type [7, 8].

$$\langle N_{\text{bond}}^{\text{ves/sol}} \rangle = z_{\text{wlsp}}^{\text{ves/sol}} \sum_{ij}^{\text{ves/sol}} \left\langle e^{-\beta U_{\text{FENE}}(r_{ij})} \right\rangle, \quad \text{(S8)}$$

where the sum runs over either all pairs of membrane vertices and anchoring points or solvent particles and anchoring points.

The wall-spring-induced attraction can be exactly precomputed and compensated by a repulsion between all particles and anchoring points that can potentially be connected by wall springs [7],

$$U_{\text{comp}}(r) = \begin{cases} k_B T \ln\left(1 - \dfrac{r^2}{r_{\max}^2}\right), & r < r_{\max}, \\ 0, & r \geq r_{\max}. \end{cases} \quad \text{(S9)}$$

Thereby, the wall-spring thermostat allows us to modulate the friction at the substrate without altering equilibrium properties. Two parameters dictate the properties of the wall-spring thermostat – the activity, z_{wlsp}, of wall springs and their average lifetime, which is dictated by the wall-spring update period, N_{wlsp} (here same for all

wall-spring types). For more details on the implementation and background, refer to Jana et al. [7].

S1.3 Soft interactions and Dissipative-Particle-Dynamics (DPD) thermostat

Hydrodynamic interactions with the explicit solvent are modeled using dissipative particle dynamics (DPD) [9, 10]. The pair interaction between the particles is a conservative, soft repulsion

$$\mathbf{F}_{ij}^{C} = \begin{cases} a_{ij}\left(1 - \frac{r_{ij}}{r_c}\right)\hat{\mathbf{r}}_{ij}, & r_{ij} < r_c, \\ 0, & r_{ij} \geq r_c, \end{cases} \tag{S10}$$

where a_{ij} is the repulsion strength, $r_{ij} = |\mathbf{r}_i - \mathbf{r}_j|$, and $\hat{\mathbf{r}}_{ij} = (\mathbf{r}_i - \mathbf{r}_j)/r_{ij}$.

The DPD thermostat consists of a random force, $\mathbf{F}_{ij}^{R}$, and a friction force, $\mathbf{F}_{ij}^{D}$. The random force represents thermal fluctuations,

$$\mathbf{F}_{ij}^{R} = \sigma\omega^{R}(r_{ij})\theta_{ij}\Delta t^{-1/2}\hat{\mathbf{r}}_{ij}, \tag{S11}$$

and $\omega^{R}(r_{ij})$ is a weighting function with finite support, $r_{ij} < r_c$. σ is the noise amplitude, θ_{ij} is a random number with zero mean and unit variance, and Δt is the timestep. The friction force is proportional to the relative velocity, $\mathbf{v}_{ij} = \mathbf{v}_i - \mathbf{v}_j$, of the particle pair ij

$$\mathbf{F}_{ij}^{D} = -\gamma\omega^{D}(r_{ij})\left(\hat{\mathbf{r}}_{ij} \cdot \mathbf{v}_{ij}\right)\hat{\mathbf{r}}_{ij}, \tag{S12}$$

where γ is the friction strength, and $\omega^{D}(r)$ is a weight function. The fluctuation-dissipation theorem relates the weight functions and amplitudes [10]

$$\omega^{D}(r) = [\omega^{R}(r)]^{2}, \tag{S13}$$

$$\sigma^{2} = 2\gamma k_B T. \tag{S14}$$

We use the standard weight function $\omega^{R}(r) = 1 - r/r_c$ for $r < r_c$ and zero otherwise. Since the DPD thermostat acts between pairs of particles within a cutoff radius, r_c, momentum is locally conserved, resulting in hydrodynamic behavior.

S1.4 Simulation parameters

Table S1: Simulation parameters used in this work. All quantities are given in reduced units where length is measured in units of r_c^{DPD}, energy in units of k_BT, and time in units of $\tau = \sqrt{m(r_c^{\mathrm{DPD}})^2/k_BT}$. Superscripts denote run-type-specific values: † non-equilibrium steady-state runs; ‡ adhesion-cycle runs.

**Parameter**	**Symbol**	**Value**
General		
Integration timestep	Δt	$0.001\ \tau$
Vesicle membrane		
Number of vertices	N_{vert}	2561
Bending rigidity (Hamiltonian)	κ	$25.0\ k_bT$
Equilibrium radius	R_0	$9.0\ r_c^{\mathrm{DPD}}$
Area per vertex	a_0	$0.52\ (r_c^{\mathrm{DPD}})^2$
Spontaneous curvature	C_0	$0.0\ (r_c^{\mathrm{DPD}})^{-1}$
Attractive–repulsive bond potential		
Equilibrium bond length	r_0	$0.8\ r_c^{\mathrm{DPD}}$
Strength	k_b	$100.0\ k_bTr_c^{\mathrm{DPD}}$
Fraction parameter 1	f_1	1/3
Fraction parameter 2	f_2	1/6
Divergence bound	δ_{div}	$0.0039\ r_c^{\mathrm{DPD}}$
Bond-flip period	τ_{flip}	$0.2\ \tau$
Wall geometry		
Bottom wall pos. (attractive)	z_{bot}	$-13.0\ r_c^{\mathrm{DPD}}$
Top wall pos. (repulsive only)	z_{top}	$+14.8\ r_c^{\mathrm{DPD}}$
Wall interactions (Mie, all particle types, both walls)		
Mie exponent (repulsive)	n	9
Mie exponent (attractive)	m	3
Interaction range	σ_w	$0.5\ r_c^{\mathrm{DPD}}$
Solvent–wall strength	ϵ_{sol}	$0.001\ k_bT$
Cutoff radius	r_c	$4.0\ r_c^{\mathrm{DPD}}$
Vesicle–wall adhesion (bottom wall only, P_ves only)		
Vesicle–wall Mie strength	ϵ_W	-2.179 / $-2.799\ k_bT^{\mathrm{a}}$
Wall springs (WLSP)		
Spring constant	k_{sp}	$4.0\ k_bT/(r_c^{\mathrm{DPD}})^2$
Maximum extension	r_{max}	$1.2\ r_c^{\mathrm{DPD}}$
Activity (W_wall–P_ves)	$z_{\mathrm{wlsp}}^{\mathrm{ves}}$	$4.0^\dagger$ / $8.0^\dagger$ / 8.0 (ph. 1&3)‡ / 4.0 (ph. 2)‡
Activity (W_wall–P_sol)	$z_{\mathrm{wlsp}}^{\mathrm{sol}}$	4.0 (all runs)

Table S1 continued...

**Parameter**	**Symbol**	**Value**
Wall-spring update period	τ_{wlsp}	0.05τ
DPD solvent		
DPD solvent density	ρ	$3.0\ (r_c^{\text{DPD}})^{-3}$
DPD cutoff radius	r_c^{DPD}	1.0
Repulsion (sol–sol)	$A_{\text{sol-sol}}$	$25.0\ k_bT/r_c^{\text{DPD}}$
Repulsion (sol–ves)	$A_{\text{sol-ves}}$	$60.0\ k_bT/r_c^{\text{DPD}}$
Repulsion (ves–ves)	$A_{\text{ves-ves}}$	$0.0\ k_bT/r_c^{\text{DPD}}$
Repulsion (wall–sol)	$A_{\text{wall-sol}}$	$25.0\ k_bT/r_c^{\text{DPD}}$
Repulsion (wall–ves)	$A_{\text{wall-ves}}$	$25.0\ k_bT/r_c^{\text{DPD}}$
Dissipation strength	γ_{DPD}	$4.5\ k_bT\tau/(r_c^{\text{DPD}})^2$
Interior heavy particles		
Sedimentation force*	F_z^{in}	$-0.05\ \ k_bT/r_c^{\text{DPD}}$
Flow		
Body force (flow scan)	$F_0^{\dagger}$	10^{-6}–$1.4 \times 10^{-4}\ k_bT/r_c^{\text{DPD}}$
Body force (cycle runs)	$F_0^{\ddagger}$	$10^{-5}\ k_bT/r_c^{\text{DPD}}$
Adhesion cycle protocol‡		
Phase 1 and 3	$\tilde{\varepsilon}_w = 4,\ z_{\text{wlsp}}^{\text{ves}} = 8$	2×10^6 steps
Phase 2	$\tilde{\varepsilon}_w = 2,\ z_{\text{wlsp}}^{\text{ves}} = 4$	5×10^6 steps

[a] The Mie strengths ϵ_* are passed to HOOMD directly. Note that ϵ_W compensates the strong effective attraction from the solvent-wall interaction acting on vesicle vertices. The dimensionless adhesion parameters $\tilde{\varepsilon}_w \approx 4$ and 2 correspond to $\epsilon_W = -2.179$ and -2.799, respectively.

S2 Supporting simulation results

S2.1 Viscosity and Shear Rate

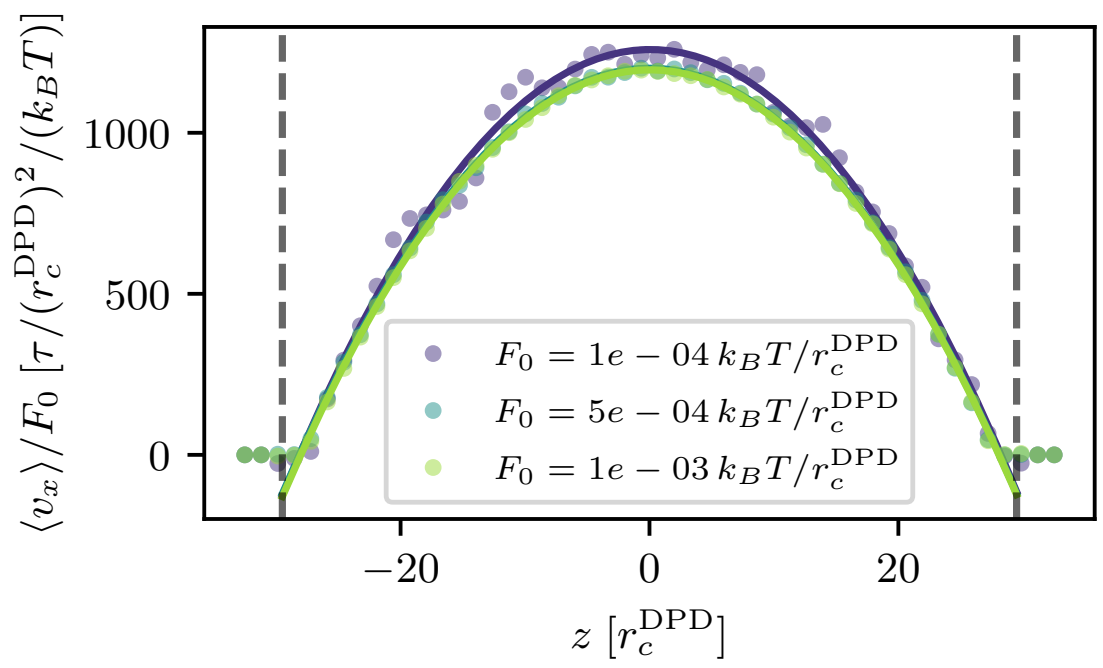

Fig. S2: Flow velocity profiles for three applied body forces in a symmetric channel. Data points show time-averaged velocity profiles $\langle v_x(z)\rangle/F$, and the solid lines are parabolic fits. The viscosity is consistent across all three forces. Using the parabolic fit, we calculate shear rates at the wall, which are used to estimate the capillary number, Ca.

The effective dynamic viscosity, $\eta = 0.97\ k_BT\tau/(r_c^{\mathrm{DPD}})^3$, of the DPD solvent was determined from the flow profile of pure solvent simulations under a uniform body force $\mathbf{F_0}$ applied to all particles. For Poiseuille flow between two parallel walls separated by a distance H, the velocity profile is parabolic.

$$\langle v_x(z)\rangle = v_{\max} - \frac{\rho F_0}{2\eta} z^2 \tag{S15}$$

where $z = 0$ corresponds to the middle of the channel, ρ is the solvent number density, and ρF_0 is the body force per unit volume. The shear rate at the wall is then obtained from the velocity gradient

$$\dot{\gamma}_{\mathrm{wall}} = \left.\frac{\mathrm{d}\langle v_x\rangle}{\mathrm{d}z}\right|_{z=z_{\mathrm{wall}}}, \tag{S16}$$

and is evaluated at $\langle v_x\rangle = 0$. For the first set of simulations, the applied forces were $< 14 \cdot 10^{-6} k_BT/r_c^{\mathrm{DPD}}$, which leads to $Ca < 1$. Hence, all observed vesicles in the simulation without buoyancy are in a regime where bending rigidity dominates, and only slight deformations appear. For simulations including buoyancy, we obtained measurements at Ca> 1, and clearly observed a much more strongly deformed vesicle.

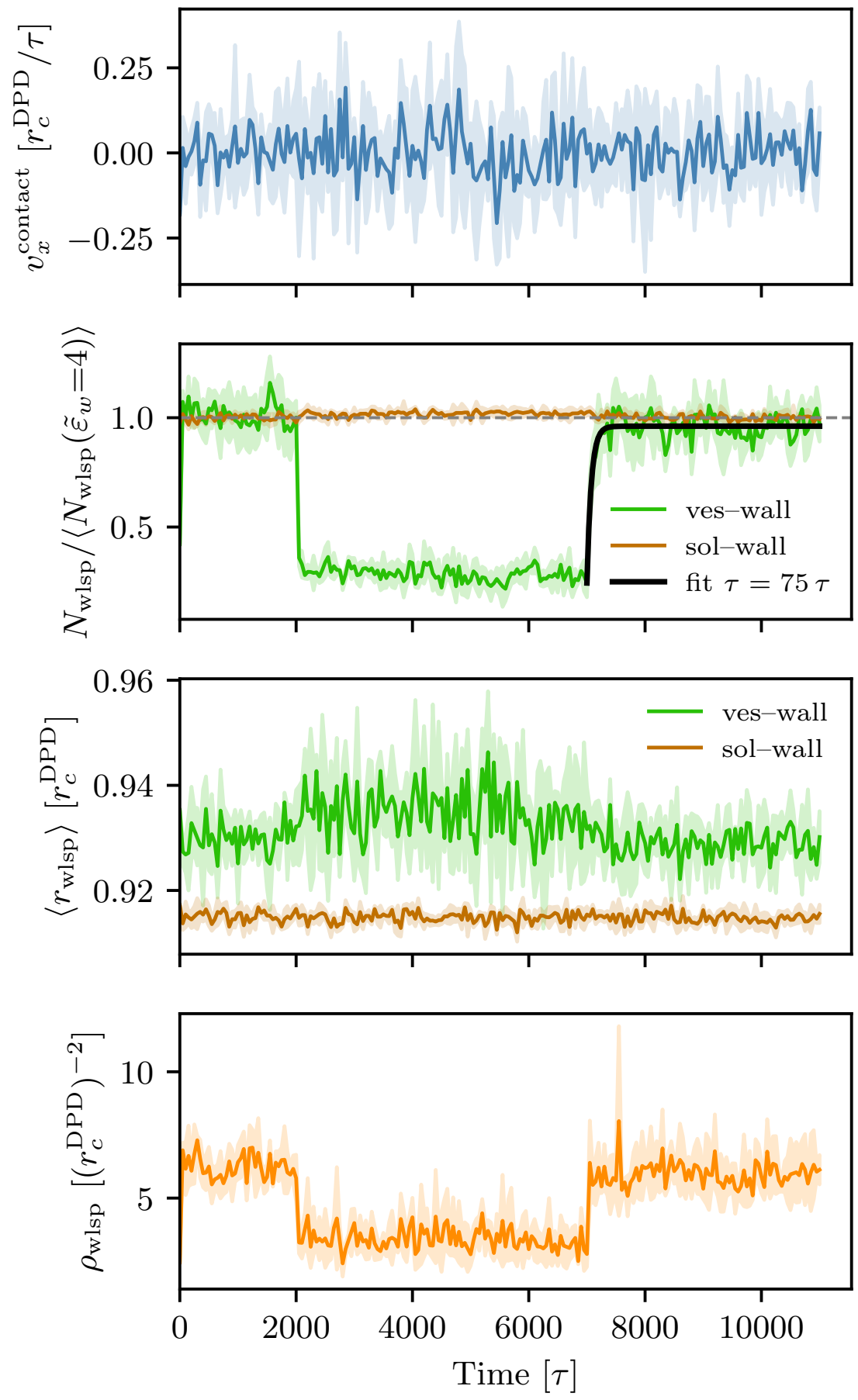

Fig. S3: Time series of adhesion dynamics during an adhesion cycle with wall-spring activity switched from $z_{\mathrm{wlsp}} = 8$ to $z_{\mathrm{wlsp}} = 4$ and back. Data are averaged over four runs. From top to bottom: (i) instantaneous velocity v_x of vesicle vertices in the contact zone, (ii) the number of wall springs connecting vesicle vertices and solvent particles to anchoring points on the substrate, (iii) average wall-spring extension $\langle r_{\mathrm{wlsp}}\rangle \pm$ std, and (iv) areal density of vesicle-wall springs ρ_{wlsp} within the contact zone.

S2.2 Wall-spring rupture and formation

Figure S3 presents the instantaneous velocity, v_x^{contact}, of the vesicle vertices in the contact zone in x-direction, the number, N_{wlsp}, of wall springs with both vesicle vertices and solvent particles, the average wall-spring length, and the areal density of wall springs within the contact zone. The data correspond to the adhesion cycle studied in the main paper.

The vanishing of v_x^{contact} of membrane vertices in the contact zone supports the tank-treading of the vesicle, independent of the adhesion. No stick-slip motion is observed in our model[11–13].

The adhesion is switched from ($\tilde{\varepsilon}_w = 4$, $z_{\mathrm{wlsp}}^{\mathrm{ves}} = 8$) to ($\tilde{\varepsilon}_w = 2$, $z_{\mathrm{wlsp}}^{\mathrm{ves}} = 4$), and back, while $z_{\mathrm{wlsp}}^{\mathrm{sol}} = 4$ stays constant. An almost immediate reduction in membrane wall-spring number follows the switch to lower activity because of the high rate grandcanonical wall-spring Monte-Carlo moves. The activity decreases by a factor of 2, whereas the number of membrane wall springs decreases by more than 3, indicating an additional structural change. A reduction in $\tilde{\varepsilon}_w$ decreases the contact area and increases the separation between membrane and substrate. Such an increased separation between membrane and substrate also correlates with the slightly longer wall springs during low adhesion. We also observe a slow decrease in $N_{wlsp}^{\mathrm{ves-wall}}$ for $t > 2000\tau$, while ρ_{wlsp} stays constant, indicating a second slower relaxation of the contact area with the global shape.

As the adhesion is increased again, the number of membrane wall springs initially rises rapidly but only later approaches its stationary high-adhesion value due to the slower relaxation of the contact area and membrane substrate distance.

The recovery of the bond number following the increase in activity can be described by a single exponential fit with a relaxation time $\tau_{\mathrm{bond}} \approx 75\,\tau$ (see Figure S3, second panel). This timescale reflects the re-expansion of the contact area after the vesicle has partially detached under reduced adhesion, and is therefore set by the dynamics of membrane shape relaxation rather than by bond kinetics alone. A natural estimate for the relevant timescale is provided by the Brochard-Lennon result [14] for the relaxation of membrane undulations,

$$\tau_{\mathrm{BL}} \sim \frac{\eta R_0^3}{\kappa}, \tag{S17}$$

where η is the solvent viscosity, R_0 the vesicle radius, and κ the bending rigidity. Inserting the simulation parameters yields $\tau_{\mathrm{BL}} \approx 28\,\tau$, within a factor of three of the measured value. Given that the Brochard-Lennon estimate applies strictly to free-membrane fluctuations and neglects the constraints imposed by wall contact and adhesion, the agreement is satisfactory and supports the interpretation that τ_{bond} is controlled by membrane mechanics.

S3 Supporting experimental observations

S3.1 Surface tension independent of [glucose]

[Glucose]	Surface tension [mN/m]
0	72.8 (set as control)
200	73.0
250	72.9
300	73.1
350	72.7
400	73.0

Table S3: Surface tension monitored over different [glucose]

S3.2 Stability and sharpness of the boundary between Flow A and Flow B over millimeter distances

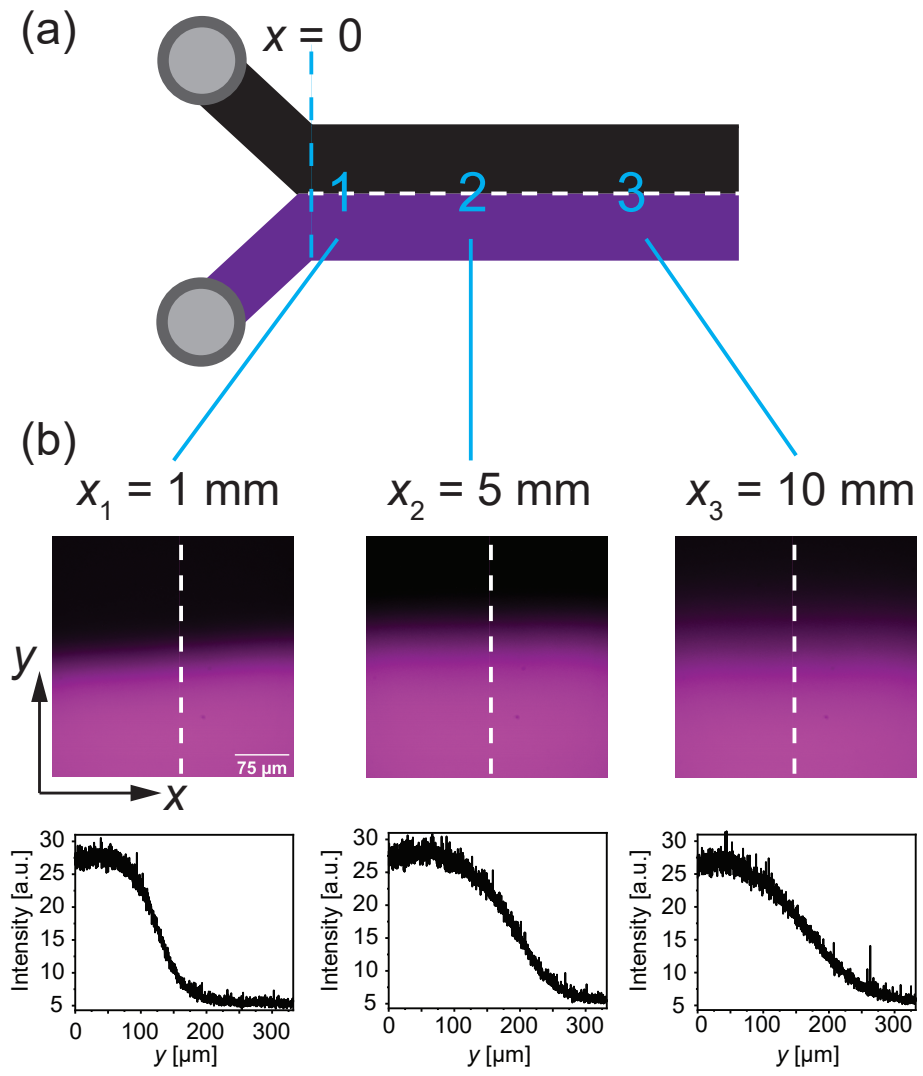

Fig. S4: Stability of boundary plotted as 3 × different positions. (a) Schematics of the experimental conditions. (b) Fluorescence images near the boundary were taken at $x = 1$, 5, and 10 mm. $x = 0$ is defined as the position of the Y-shaped junction. The line profile along the broken line at each position is presented in the bottom row.

S3.3 Confirmation of successful switching from flow A to flow B by fluorescence microscopy

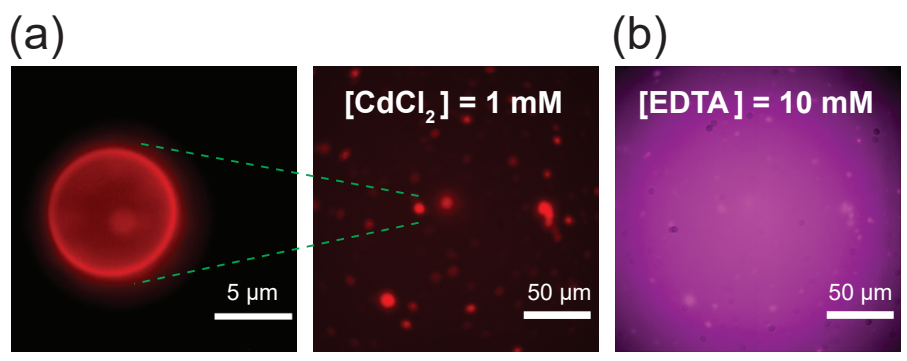

Fig. S5: (a) Fluorescence image of GUVs exposed to $[CdCl_2]$ = 1 mM (Flow A). (b) Same region of interest after varying the ratio of flow rates. EDTA-containing buffer labeled with Cy5 confirmed that GUVs are exposed to EDTA (Flow B).

S3.4 Asymmetric vesicle shape of adhered vesicle at higher flow rate

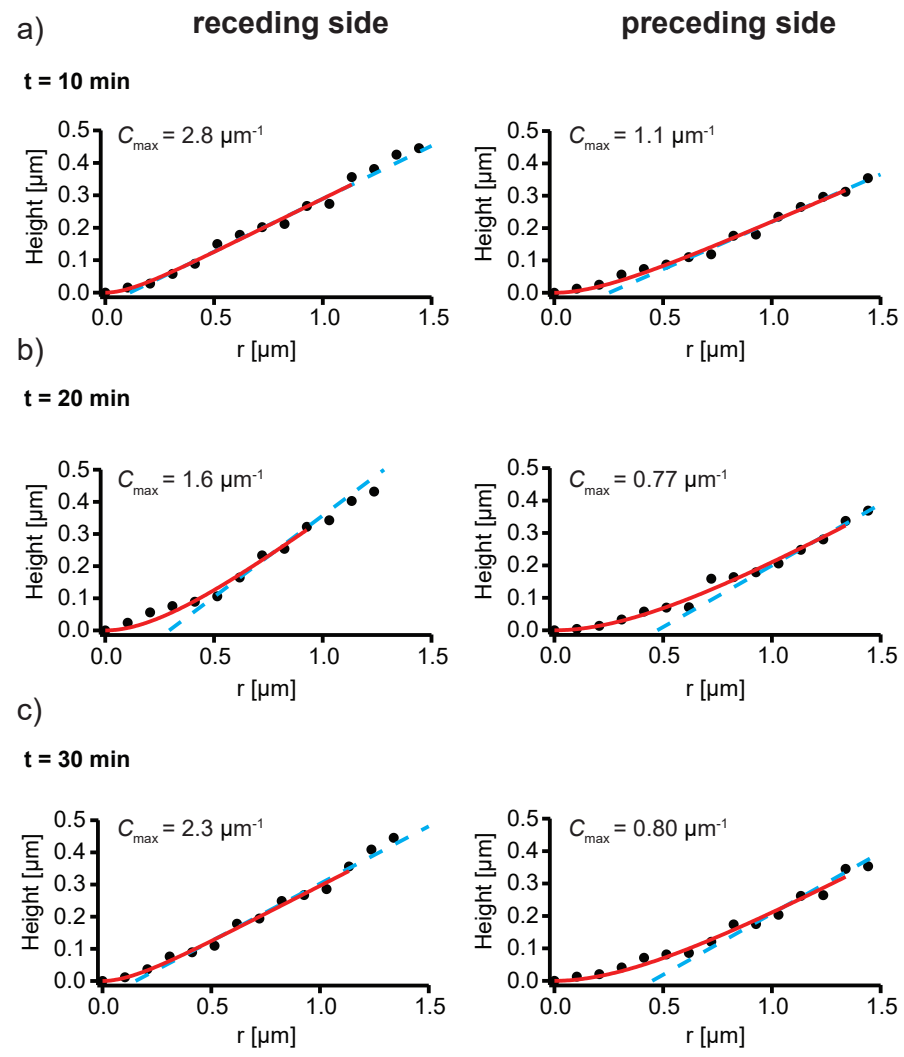

Fig. S6: Adhered vesicle under increased flow, where flow A and flow B are mixed at a ratio of 160 μL min^{-1}: 30 μL min^{-1}. The same vesicle was monitored over 30 min exposed to $[CdCl_2]$ = 1 mM, and the height profiles and corresponding curvatures C_{max} were monitored at a) $t = 10$ min, b) $t = 20$ min and c) $t = 30$ min after turning the flow on.

S3.5 Varying vesicle adhesion without flow

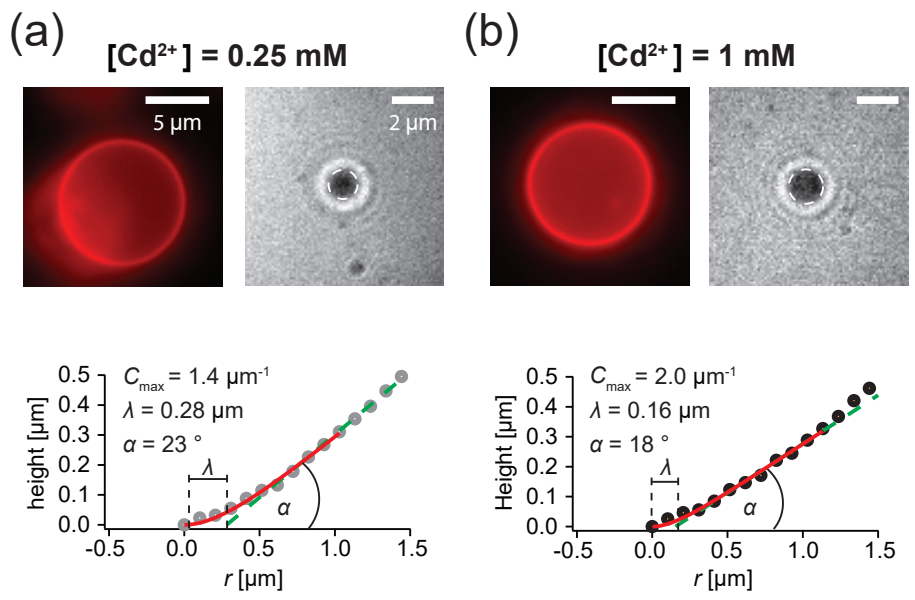

Fig. S7: Vesicles under steady conditions (no flow). (a) Fluorescence and RICM images of GUVs adhered to pAA-Cys5 substrates in the presence of $[CdCl_2] = 0.25$ mM. Reconstructed height profile with the key parameters. (b) Fluorescence and RICM images in the presence of $[CdCl_2] = 1$ mM, and the corresponding height profile.

References

[1] Helfrich, W.: Elastic Properties of Lipid Bilayers: Theory and Possible Experiments. Zeitschrift für Naturforschung C **28**(11-12), 693–703 (1973) https://doi.org/10.1515/znc-1973-11-1209

[2] Meyer, M., Desbrun, M., Schröder, P., Barr, A.H.: Discrete Differential-Geometry Operators for Triangulated 2-Manifolds. In: Hege, H.-C., Polthier, K. (eds.) Visualization and Mathematics III, pp. 35–57. Springer, Berlin, Heidelberg (2003). https://doi.org/10.1007/978-3-662-05105-4_2

[3] Gueguen, G., Destainville, N., Manghi, M.: Fluctuation tension and shape transition of vesicles: Renormalisation calculations and Monte Carlo simulations. Soft Matter **13**(36), 6100–6117 (2017) https://doi.org/10.1039/C7SM01272A

[4] Gompper, G., Kroll, D.M.: Triangulated-Surface Models of Fluctuating Membranes, 2nd edn., pp. 359–426. WORLD SCIENTIFIC, Singapore (2004). https://doi.org/10.1142/9789812565518_0012

[5] Noguchi, H., Gompper, G.: Dynamics of fluid vesicles in shear flow: Effect of membrane viscosity and thermal fluctuations. Physical Review E **72**(1), 011901 (2005) https://doi.org/10.1103/PhysRevE.72.011901

[6] Vutukuri, H.R., Hoore, M., Abaurrea-Velasco, C., van Buren, L., Dutto, A., Auth, T., Fedosov, D.A., Gompper, G., Vermant, J.: Active particles induce large shape deformations in giant lipid vesicles. Nature **586**(7827), 52–56 (2020) https://doi.org/10.1038/s41586-020-2730-x

[7] Jana, P.K., Bacova, P., Schneider, L., Kobayashi, H., Hollborn, K.-U., Polinska, P., Burkhart, C., Harmandaris, V.A., Müller, M.: Wall-spring thermostat: A novel approach for controlling the dynamics of soft coarse-grained polymer fluids at surfaces. Macromolecules **55**(13), 5550–5566 (2022)

[8] Chappa, V.C., Morse, D.C., Zippelius, A., Müller, M.: Translationally Invariant Slip-Spring Model for Entangled Polymer Dynamics. Phys. Rev. Lett. **109**(14), 148302 (2012) https://doi.org/10.1103/PhysRevLett.109.148302

[9] Hoogerbrugge, P.J., Koelman, J.M.V.A.: Simulating Microscopic Hydrodynamic Phenomena with Dissipative Particle Dynamics. Europhysics Letters **19**(3), 155 (1992) https://doi.org/10.1209/0295-5075/19/3/001

[10] Español, P., Warren, P.: Statistical Mechanics of Dissipative Particle Dynamics. Europhysics Letters **30**(4), 191 (1995) https://doi.org/10.1209/0295-5075/30/4/001

[11] Blass, J., Albrecht, M., Bozna, B.L., Wenz, G., Bennewitz, R.: Dynamic effects in friction and adhesion through cooperative rupture and formation of supramolecular bonds. Nanoscale **7**(17), 7674–7681 (2015)

[12] Sabass, B., Schwarz, U.S.: Modeling cytoskeletal flow over adhesion sites: competition between stochastic bond dynamics and intracellular relaxation. Journal of Physics: Condensed Matter **22**(19), 194112 (2010)

[13] King, M.R., Hammer, D.A.: Multiparticle adhesive dynamics. interactions between stably rolling cells. Biophysical journal **81**(2), 799–813 (2001)

[14] Brochard, F., Lennon, J.F.: Frequency spectrum of the flicker phenomenon in erythrocytes. Journal de Physique **36**(11), 1035–1047 (1975) https://doi.org/10.1051/jphys:0197500360110103500
